# The virulome of *Streptomyces scabiei* in response to cello-oligosaccharide elicitors

**DOI:** 10.1099/mgen.0.000760

**Published:** 2022-01-17

**Authors:** Benoit Deflandre, Nudzejma Stulanovic, Sören Planckaert, Sinaeda Anderssen, Beatrice Bonometti, Latifa Karim, Wouter Coppieters, Bart Devreese, Sébastien Rigali

**Affiliations:** ^1^​ InBioS-Centre for Protein Engineering, Institut de Chimie B6a, University of Liège, B-4000, Liège, Belgium; ^2^​ Laboratory for Microbiology, Department of Biochemistry and Microbiology, Ghent University, B-9000, Ghent, Belgium; ^3^​ Genomics Platform, GIGA, University of Liège, B-4000, Liège, Belgium

**Keywords:** common scab disease, plant-host interaction, plant colonization, plant pathogen, metabolomics, biosynthetic gene cluster

## Abstract

The development of spots or lesions symptomatic of common scab on root and tuber crops is caused by few pathogenic *Streptomyces* with *Streptomyces scabiei* 87–22 as the model species. Thaxtomin phytotoxins are the primary virulence determinants, mainly acting by impairing cellulose synthesis, and their production in *S. scabiei* is in turn boosted by cello-oligosaccharides released from host plants. In this work we aimed to determine which molecules and which biosynthetic gene clusters (BGCs) of the specialized metabolism of *S. scabiei* 87–22 show a production and/or a transcriptional response to cello-oligosaccharides. Comparative metabolomic analyses revealed that molecules of the virulome of *S. scabiei* induced by cellobiose and cellotriose include (i) thaxtomin and concanamycin phytotoxins, (ii) desferrioxamines, scabichelin and turgichelin siderophores in order to acquire iron essential for housekeeping functions, (iii) ectoine for protection against osmotic shock once inside the host, and (iv) bottromycin and concanamycin antimicrobials possibly to prevent other microorganisms from colonizing the same niche. Importantly, both cello-oligosaccharides reduced the production of the spore germination inhibitors germicidins thereby giving the ‘green light’ to escape dormancy and trigger the onset of the pathogenic lifestyle. For most metabolites - either with induced or reduced production - cellotriose was revealed to be a slightly stronger elicitor compared to cellobiose, supporting an earlier hypothesis which suggested the trisaccharide was the real trigger for virulence released from the plant cell wall through the action of thaxtomins. Interestingly, except for thaxtomins, none of these BGCs’ expression seems to be under direct control of the cellulose utilization repressor CebR suggesting the existence of a yet unknown mechanism for switching on the virulome. Finally, a transcriptomic analysis revealed nine additional cryptic BGCs that have their expression awakened by cello-oligosaccharides, suggesting that other and yet to be discovered metabolites could be part of the virulome of *S. scabiei *.

## Data Summary

RNAseq data were publicly deposited, and our experimental and analytical pipeline were described on the GEO database repository (accession number: GSE181490)

The authors confirm all supporting data, code and protocols have been provided within the article or through supplementary data files.

**Impact Statement**

Unveiling the environmental triggers that signal proper conditions for host colonization and what is the composition of the arsenal of metabolites specialized for this task (the virulome) is key to understand host-pathogen interactions. In this work, focused on the induction of common scab caused by *Streptomyces* species, we provided further knowledge to both aspects i.e. (i) highlighting cellotriose as the best environmental trigger of the pathogenic lifestyle, and ii) identifying the set of metabolites that specifically respond to cello-oligosaccharides emanating from the plant under attack and that may be part of the virulome along with the thaxtomin phytotoxins. Importantly, we also revealed that the expression of nine cryptic/orphan biosynthetic gene clusters (BGCs) involved in the production of unknown compounds was activated upon cello-oligosaccharides import suggesting that a significant part of the virulome of *S. scabiei* remains to be discovered. Finally, we unexpectedly found that the expression control of most of the known and cryptic BGCs does not depend on the cello-oligosaccharide utilization repressor CebR which suggests the existence of another and yet unknown mechanism for the onset of pathogenicity in *S. scabiei *.

## Introduction

*Streptomyces scabiei* (synonym *Streptomyces scabies *) is responsible for causing the disease called ‘common scab’ (CS) on root and tuber crops. Together with a dozen other phylogenetically related *Streptomyces* species, *S. scabiei* colonizes and infects underground storage organs like potato tubers, beets, radishes, turnips, carrots, and peanuts [1, 2]. CS lesions cause significant economic losses throughout the world, with potato being the most affected crop. The range of symptoms and lesion morphologies on potato tubers goes from superficial to raised or deep-pitted scabs [3]. Although CS is characterized by skin defects, it mostly affects the visual aspect of the tuber tissues and root, sometimes also reducing their size, causing a significant drop in the quality and marketability of the potato tubers [3].

The virulence factors which predominantly contribute to the development of CS are the thaxtomin phytotoxins which are nitrated diketopiperazines [4]. To date, eleven thaxtomin analogues have been identified, thaxtomin A being the predominant form associated with the disease [5–7]. While the molecular targets are still unknown, thaxtomin A alters the expression of host genes involved in cellulose biosynthesis, cell wall remodelling and strengthening [8, 9]. Cellulose synthase complexes were also shown to be affected in their density and motility [8], possibly due to endocytosis triggered by thaxtomin A [10]. *In vivo*, thaxtomin A causes multiple symptoms to plant targets including necrosis, perturbation of ion fluxes, cell hypertrophy, callose deposition, and ectopic lignin formation [11]. In addition, wounded or immature sites are affected, inducing synthesis of hemicellulose and pectins and leading to the deposition and excessive accumulation of layers of periderm [12].

Apart from thaxtomins, other specialized metabolites are known or hypothetized to play important roles in plant colonization and infection by *S. scabiei *. Several studies revealed that concanamycins and coronafacoyl phytotoxins also contribute to the development of plant disease [11]. In synergy with thaxtomins, concanamycins were shown to play an essential role in the type and morphology of developed lesions [13]. Contrary to concanamycins and thaxtomins, the exact roles of coronafacoyl phytotoxins in CS disease development are still relatively vague and were shown to be non-essential for pathogenicity development [11]. However, their impact via modulating jasmonate hormone signalling networks could assist in overcoming host defence mechanisms [14]. N-coronafacoyl-l-isoleucine (CFA-l-Ile) is the major product of the coronafacoyl gene cluster [15]. A wide spectrum of virulence-associated activities of CFA-l-Ile, like tissue hypertrophy, leaf chlorosis and inhibition of root elongation, were reported in plants. Nevertheless, coronafacoyl phytotoxins are found in non-pathogenic *Streptomyces* spp. as well, suggesting some additional unidentified roles along with the already cited disease-related activities [16]. Recently, the production of two novel phytotoxic metabolites was highlighted in *S. scabiei *. Rotihibins C and D are lipopeptides that significantly reduce the photochemistry efficiency of the photosystem II which in turn affects the growth of *Arabidopsis thaliana* and *Lemna minor* at low concentrations. At even lower concentrations, *L. minor* plantlets instead exhibit an increase in their surface area, suggesting a hormetic effect of rotihibins [17].

Siderophores are also key metabolites for host infecting bacteria, iron being indispensable for housekeeping functions such as DNA replication and protein synthesis. Next to desferrioxamines that are essential for *Streptomyces* survival in iron limited environments [18], *S. scabiei* and related species have the ability to produce diverse and specific siderophores including scabichelin and turgichelin [19], as well as pyochelin [20] together with three other yet unknown iron chelators deduced from genome mining analysis. This multitude of siderophores with high affinity for iron would guarantee *S. scabiei* to capture iron trapped in its hosts. However, although iron acquisition might contribute to the onset of pathogenicity, *in planta* bioassays showed that there is no connection between virulence and pyochelin production by *S. scabiei* [20].

How *S. scabiei* senses the presence of its plant host and triggers its specialized metabolism required for virulence has also been a main research topic. Induction of thaxtomin biosynthesis requires the import of cello-oligosaccharides (cellobiose and/or cellotriose) by the sugar ABC transporter composed by CebE as the sugar-binding component, CebF and CebG as components of the membrane permease, and MsiK to provide energy to the transport via ATP hydrolysis [21–23]. Once inside the cytoplasm, the imported cello-oligosaccharides inhibit the DNA-binding ability of the transcriptional repressor CebR which in turn allows the expression of the *txt* cluster pathway-specific activator TxtR [22, 24].

Although the path from cello-oligosaccharide uptake to activation of thaxtomin biosynthesis is well described at the molecular level, many questions remain unsolved. The first issue regards cellobiose itself as a natural elicitor of CS disease. Most *in vivo* and *in vitro* studies on the induction of the pathogenic lifestyle of *S. scabiei* used cellobiose and not cellotriose as the triggering factor. The main reason why cellotriose is usually excluded from laboratory studies is because it is much more expensive and less available in large quantities compared to cellobiose. However, incubation of tobacco and radish seedlings with thaxtomin A showed release of cellotriose but not cellobiose [25]. The disaccharide on the other hand, is the main product of cellulose hydrolysis by the cellulolytic system, which naturally occurs upon organic matter turnover by the soil microflora. However, the ability of *S. scabiei* to degrade cellulose is insignificant despite possessing a complete cellulolytic system [25–27]. While the molecular mechanism silencing the cellulolytic system of *S. scabiei* is unknown, it avoids the release of cellobiose from decaying plant biomass, hence preventing this bacterium to be a protagonist in the mineralization of organic soils. This particularity could be a major evolutionary adaptation that somehow ‘forces’ *S. scabiei* to colonize living plant tissues instead. Sensing cellotriose released by the depolymerization of cellulose caused by thaxtomin, together with silencing the cellulolytic system and thus avoiding the release of cellobiose, could allow this bacterium to discriminate if cellulose by-products originate from living or instead dead plant cell walls [25, 27]. The hypothesis that the trisaccharide would indeed be the real elicitor of host colonization is also supported by the higher production yields of thaxtomin A when *S. turgidiscabies* and *S. acidiscabies* were cultivated with cellotriose than with cellobiose [25]. In line with this observation, we also showed that CebE - the protein of the cello-oligosaccharide importer of *S. scabiei* - has a very high affinity for the trisaccharide, even higher than for cellobiose, which is an unusual feature for sugar ABC transporters in streptomycetes [23]. Also, once imported, cellotriose can inhibit the repressor activity of CebR in two ways, i.e. i) directly by itself as allosteric effector of CebR, and ii) indirectly via cellobiose resulting from its hydrolysis by the beta-glucosidase BglC [22, 28]. Therefore, when cellotriose is imported inside the cytoplasm, it could well be that the cellobiose resulting from its hydrolysis by BglC would be mostly responsible for the onset of thaxtomin production, since the disaccharide is the best allosteric inhibitor of CebR [22] and also a co-activator of TxtR [24].

An even more important question that remains to be solved is the exact composition of the arsenal of specialized metabolites that constitute the virulome of *S. scabiei *. Are thaxtomins the only phytotoxins that respond to virulence elicitors or do other specialized metabolites display the same production response? Recently, the group of Professor Dawn Bignell has shown that cultivation of *S. scabiei* on oat bran agar (OBA) medium is not only able to induce thaxtomin production, but also other specialized metabolites known or predicted to play an important role in colonizing and infecting the plant host tissues, i.e. CFA-l-Ile, concanamycins, siderophores (desferrioxamines and pyochelin), and the indole-3-acetic acid auxin (IAA) [29]. OBA is a complex plant-based medium in which cello-oligosaccharides are proposed to be responsible for the induction of thaxtomin production [25]. However, it cannot be excluded that some of the other compounds present in OBA influence – positively or negatively, alone or in combination – the production of specialized metabolites.

As previous studies suggested cellobiose and/or cellotriose to be natural elicitors of the pathogenic response of *S. scabiei* [27], their specific contribution to the induction of the metabolome requires further investigation. In this work we provide answers to whether cellotriose can – equally to cellobiose – trigger the ‘virulome’ of *S. scabiei* and if the cello-oligosaccharide mediated induction takes place at the transcriptional level. Our work revealed that cellotriose is a better inducer of the virulome of *S. scabiei* compared to cellobiose. Our transcriptomic analysis also shows that cryptic/orphan biosynthetic gene clusters have their expression awakened by cello-oligosaccharides suggesting that yet unknown metabolites would be part of the virulome of *S. scabiei *.

## Methods

### Strain and culture conditions

*Streptomyces scabiei* 87–22 and its Δ*cebR* mutant [22] were routinely cultured in Tryptic Soy broth (TSB, 30 g l^−1^, Sigma-Aldrich) or ISP2 (for 1 l: 4 g Yeast Extract, 10 g Malt Extract, 4 g Dextrose, pH 7.2) liquid media at 28 °C under shaking (180 r.p.m., New Brunswick Innova 44 incubator shaker). Modified thaxtomin defined medium (TDM) ([25], without l-Sorbose) was used as minimal medium, supplemented with maltose 0.5 % (Sigma-Aldrich) in which cellobiose (Carbosynth) and cellotriose (Megazyme) were added as inducers. Sucrose was purchased from Merck.

### Transcriptomics

### Cultures and sampling

Pre-cultures of *S. scabiei* 87–22 (WT) and Δ*cebR* were conducted in 50 ml ISP2 medium inoculated with 4×10^7^ spores for 24 h. The mycelium was collected by centrifugation (3500 ***g*** for 5 min at room temperature (RT)) and washed twice with 20 ml TDM medium without carbon source. The mycelium was then resuspended in TDM +maltose 0.5 % (TDMm) or ISP2 to a density of 16 mg ml^−1^ (wet biomass) and then split into three Erlenmeyer flasks (per strain and culture condition) containing a culture volume of 25 ml. After 30 min of incubation in TDMm at 28 °C, a first sample (=time points 0) of 2.5 ml was collected from each flask and cellobiose or cellotriose were added to a final concentration of 2.5 mM, each into three flasks. The next samples were collected following the same procedure, 1 and 2 h (time points 1 and 2, respectively) post-addition of cello-oligosaccharides. For the ISP2 cultures containing the *S. scabiei* WT and Δ*cebR*, 2.5 ml samples were collected from each flask after 3 h of culture. All samples were collected in 15 ml Falcon tubes and centrifuged for 3 min at 3500 ***g*** (RT). The supernatant was quickly and thoroughly removed, and the tubes were immediately flash-frozen in liquid nitrogen. The frozen cell pellets were stored in a −80 °C freezer until RNA extraction.

### RNA preparation

The RiboPure Bacteria RNA Purification Kit (Invitrogen) was used for total RNA extraction. The RNAwiz lysis buffer was added to the frozen mycelium pellets and the procedure was followed according to the manufacturer’s guidelines except the bead-beating step that was extended to 20 min. The quantification and quality control of total RNA samples were performed on a Bioanalyzer 2100 (Agilent). Ribosomal RNA depletion and library preparation were carried out using the Ovation Complete Prokariotic RNAseq kit (NuGEN). The libraries were sequenced on a NextSeq 500 System (Illumina) HM 2×75 bp read length with seven million reads per library.

### Read mapping and differential expression

Sequenced reads were quality-checked and trimmed where necessary, using the Trimmomatic Software [30]. Reads were subsequently mapped to the reference genome (* S. scabiei* 87–22), using Bowtie2 [31, 32], and an average of 98.7 % of reads were aligned. For each transcript, the number of mapped reads were compiled with featureCounts [33], generating a count table on which the rest of the analysis is based. Differential expression analysis was performed in R, with the DESeq2 package [34]. RNAseq data were publicly deposited, and our experimental and analytical pipeline were described in the GEO database repository (accession number: GSE181490)

### Metabolomics

After 45 h of pre-culture in TSB inoculated with 2×10^7^ spores of *S. scabiei* 87–22, the mycelium was collected by centrifugation (3500 ***g*** for 5 min at RT) and washed twice with 20 ml TDM medium without carbon source. The washed mycelium was resuspended to a density of 200 mg ml^−1^ (wet biomass) and 1 ml was used to inoculate 25 ml plates as overlay. Three conditions were tested with three biological replicates: TDMm +cellobiose 2.5 mM; TDMm +cellotriose 2.5 mM; TDMm +sucrose 2.5 mM. After 96 h of incubation at 28 °C, half of each plate was extracted with mQ H_2_O (v/v), dried, resuspended in 1 ml of mQ H_2_O, and filtered through 0.22 µm syringe-driven filters. These metabolic extracts were diluted 20 times in 97/3/0.1 H_2_O/ACN/HCOOH to improve chromatographic and mass spectrometric performance.

The µLC-MS/MS system consisted of a Waters NanoAcquity M-Class UPLC coupled to a Waters Xevo TQ-S triple quadrupole mass spectrometer fitted with an IonKey/MS^TM^ source (Waters, MA, USA). Mobile phase A was 0.1 % formic acid in H_2_O (Biosolve) and mobile phase B was 0.1 % formic acid in acetonitrile (Biosolve). The strong and weak solutions used to wash the auto-sampler were 0.1 % HCOOH in H_2_O and 0.1 % HCOOH in acetonitrile/water/isopropanol (Biosolve) (50 : 25 : 25, v/v/v), respectively. The samples were directly injected (5 µl injection volume) to a Waters 150 µm × 100 mm, 1.8 µm HSS T3, iKey^TM^ separation device. The metabolites were eluted from the analytical column using the following gradient: 0–10 min: 3–50 % B, 10–11 min: 50–80 % B, 11–15 min: 80 % B, 15–16 min 80–3 % B, 16–25 min: 3 % B at a flow rate of 2 µl min^−1^. The column was operated at 45 °C, ionization was performed in positive mode using a voltage of 3.65 kV. The cone and collision voltage were set respectively at 35 V and 30 V, and the source temperature was 120 °C.

Detection was obtained by MRM mode with transitions of the analytes of interest and their specific retention window (±0.5 min). Selection of these transitions was based on information in the GNPS public spectral library, literature survey, optimization experiments and own findings [17] (Table S1, available in the online version of this article). Data acquisition was performed by MassLynx 4.2 software, and the data were subjected to a Savitzky-Golay smoothing in Skyline v21 [35]. The Area Under the Curve (AUC) of ion peaks was calculated and normalized to the TDMm +sucrose condition for each metabolite. Each complete set of different conditions/biological replicates was randomly analysed and repeated separately (triplicates).

### Genome mining

AntiSMASH (antibiotics and secondary metabolites analysis shell; version 5.1.2), available at https://antismash.secondarymetabolites.org, was used for genome mining [36] in combination with the internal MIBiG 2.0 (Minimum Information about a Biosynthetic Gene cluster) database [37]. The complete genome sequence of *Streptomyces scabiei* 87–22 (Ref NC_013929) was used for the prediction of BGCs. Manual inspection was carried out to rectify the synteny values provided by AntiSMASH, only considering protein sequences sharing a minimum of 60 % of identity on at least 70 % of sequence coverage. Contiguous but obviously distinct BGCs were manually split into individual BGCs, and additionally corrected as supported by literature survey.

## Results

### The specialized metabolism of *S. scabiei* 87-22

Prior to assessing the transcriptomic and metabolomic responses of *S. scabiei* 87–22 to virulence elicitors, we updated the current knowledge on the BGCs of the specialized metabolism of this species. A genome mining analysis has recently been performed by Liu *et al*. [29], identifying 34 BGCs including eight terpenes, six non-ribosomal peptide synthetases (NRPSs), six polyketide synthases (PKSs), one hybrid PKS-NRPS BGC, five ribosomally synthesized and post-translationally modified peptides (RiPPs), four siderophores, and four other types of BGCs (betalactone, butyrolactone, melanin, and ectoine). We performed additional and manual rounds of inspection (additional blast searches and a literature survey) in order to (i) identify possible BGC delimitation issues and correct BGC length, (ii) split individual BGCs into multiple BGCs, and (iii) identify BGCs involved in the production of known natural products absent from the MIBiG database (version 2.0). In total, 12 other BGCs were identified through these additional steps leading to a final list of 46 BGCs (Table 1). Among these 12 additional BGCs, there was only one BGC for which the natural product is known, namely BGC#33b coding for melanin. The other 11 BGCs, including BGC#1b (NRPS), BGC#6b (terpene), BGC#7b (bacteriocin), BGC#16b (lanthipeptide), BGC#23b (butyrolactone), BGC#23 c linear azole/azoline-containing peptide (LAP), BGC#23d (PKS), BGC#27 a (Type 1 PKS), BGC#29 a (Type 3 PKS) and BGC#31b (linaridin) displayed relatively low similarity levels with genes of BGCs associated with the biosynthesis of known compounds (Table 1).

**Table 1.** Prediction of BGCs involved in specialized metabolite production in *Streptomyces scabiei* 87–22

BGC	BGC genes [old locus tag]	BGC length (bp)	Product type	Specialized metabolite	Bioactivity	Most similar BGC (%) Species	Ref / MIBiG ID
1a	SCAB_RS00610-00670 [SCAB_1361–1481]	26 675	Siderophore	Pyochelin	Iron uptake	Pyochelin (100) * S. scabiei* 87–22	[20]
1b	SCAB_RS00675-00760 [SCAB_1491–1671]	18 891	NRPS	Cryptic	Unknown	None	na
2	SCAB_RS00870-01005 [SCAB_1951–2231]	34 118	Betalactone	Cryptic	Unknown	*Esmeraldin (4*) * S *. *antibioticus *	BGC0000935
3	SCAB_RS01465-01545 [SCAB_3221–3351]	32 892	NRPS	Rothibins	Plant growth inhibitory effect	Rothibins (100) * S. scabiei* RL-34	[17]
4	SCAB_RS01655-01700 [SCAB_3601–3671]	15 765	Lanthipeptide	Cryptic	Unknown	None	na
5	SCAB_RS02290-02370 [SCAB_4951–5131]	19 986	Terpene	2-methylisoborneol	Smell of soil	2-methylisoborneol (100) * S. griseus *	[53]
6a	SCAB_RS02505-02545 [SCAB_5421–5511]	11 090	Terpene	Isorenieratene	Light harvesting photoprotection	Isorenieratene (100) *S. argillaceus*	BGC0001456
6b	SCAB_RS02550-02590 [SCAB_5521–5601]	7346	Terpene	Cryptic	Unknown	Guadinomine (4) *S*. *sp*. K01-0509	BGC0000998
7a	SCAB_RS04050-04080 [SCAB_8601–8661]	13 988	Lanthipeptide	Informatipeptin	Antimicrobial	Informatipeptin (63) * S. viridochromogenes* DSM 40736	BGC0000518
7b	SCAB_RS04085-04095 [SCAB_8681–8701]	3358	Bacteriocin	Cryptic	Unknown	None	na
8	SCAB_RS05720-05745 [SCAB_12041–12091]	6531	Butyrolactone	Cryptic	Unknown	Pyocyanine (14) *P. aeruginosa* PAO1	BGC0000936
9	SCAB_RS06125-06180 [SCAB_12881–13001]	13 913	Terpene	Hopene	Protection against water loss	Hopene (92) * S. coelicolor* A3(2)	[54]
10	SCAB_RS08670-08720 [SCAB_18341–18441]	11 906	Siderophore	Cryptic	Unknown	Grincamycin (9) * S. lusitanus *	BGC0000229
11 a	SCAB_RS09255-09340 [SCAB_19561–19741]	27 673	NRPS-like	Cryptic	Unknown	Stenothricin (11) * S. filamentosus* NRRL 15998	BGC0000431
11b	SCAB_RS09350-09410 [SCAB_19761–19891]	11 446	NRPS-like	Cryptic	Unknown	s56-p1 (43) *S*. cp. Soc090715ln-17	BGC0001764
12	SCAB_RS09510 [SCAB_20121]	2207	Terpene	Geosmin	Earthy odorant	Geosmin (100) * S. coelicolor* A3(2)	[55]
13	SCAB_RS09780-09830 [SCAB_20701–20801]	10 413	Bacteriocin	Cryptic	Unknown	None	na
14	SCAB_RS10905-10995 [SCAB_23071–23271]	20 828	Terpene	Cryptic	Unknown	FD-594 (7) *S*. sp. Ta-0256	BGC0000222
15	SCAB_RS11635-11660 [SCAB_24651–24711]	9975	Siderophore	Cryptic	Unknown	None	na
16 a	SCAB_RS15070-15100 [SCAB_31761–31841]	18 264	NRPS	Thaxtomins	Phytotoxin	Thaxtomin A (100) * S. scabiei* 87–22	[56]
16b	SCAB_RS15145-15225 [SCAB_31961–32131]	19 409	Lanthipeptide	Cryptic	Unknown	None	na
17	SCAB_RS20585-20630 [SCAB_43271–43361]	9782	Type 2 PKS	Spore pigment	Pigment	Spore pigment (75) * S. avermitilis *	BGC0000271
18	SCAB_RS20845-20995 [NA-44151]	42 317	NRPS, Type 1 PKS	Cryptic	Unknown	None	na
19	SCAB_RS22630-22710 [SCAB_47531–47711]	21 090	Lanthipeptide	Cryptic	Unknown	None	na
20	SCAB_RS26995-27050 [SCAB_56591–56711]	17 166	Bacteriocin, bottromycin	Bottromycins	Antibacterial	Bottromycin A2 (100) * S. scabiei* 87–22	[57]
21	SCAB_RS27660-27675 [SCAB_57921–57951]	5032	Siderophore	Desferrioxamines	Iron uptake	Desferrioxamines (100) *S*. *sp*. Id38640	BGC0001478
22	SCAB_RS28265-28270 [SCAB_59231–59241]	1290	Melanin	Melanin	Pigment	Melanin (100) * S. griseus *	[58]
23 a	SCAB_RS30025-30125 [SCAB_62881–63081]	30 803	Type 1 PKS	Cryptic	Unknown	None	na
23b	SCAB_RS30085-30160 [NA-63151]	23 408	Butyrolactone	Cryptic	Unknown	None	na
23 c	SCAB_RS30120-30205 [NA-63271]	24 765	LAP	Cryptic	Unknown	None	na
23d	SCAB_RS30125-30265 [SCAB_63081–63401]	37 334	PKS-like	Cryptic	Unknown	None	na
24	SCAB_RS33835-33850 [SCAB_70711–70741]	3166	Ectoine	Ectoine	Osmoprotectant	Ectoine (100) * S. scabiei* 87–22	[59]
25	SCAB_RS34860-34975 [SCAB_72851–73081]	24 960	NRPS-like	Cryptic	Unknown	None	na
26	SCAB_RS35245-35325 [SCAB_73651–73801]	17 977	Terpene	Cryptic	Unknown	None	na
27 a	SCAB_RS37770-37840 [SCAB_78881–79041]	23 685	Type 1 PKS	Cryptic	Unknown	None	na
27b	SCAB_RS37860-37955 [SCAB_79081–79081]	27 749	Indole	Cryptic	Unknown	5-isoprenylindole-3-carboxylate β-d-glycosyl ester (14) *S*. Sp. RM-5–8	BGC0001483
28	SCAB_RS38095-38165 [SCAB_79581–79721]	31 375	Type 1 PKS	Coronafacoyl phytotoxins	Phytotoxin	Coronafacoyl phytotoxin, (100) * S. scabiei* 87–22	[60]
29 a	SCAB_RS38310-38370 [SCAB_80021–80131]	15 153	Type 3 PKS	Cryptic	Unknown	Daptomycin (11) * S. filamentosus* NRRL 11379	BGC0000336
29b	SCAB_RS38390 [SCAB_80171]	1184	Type 3 PKS	Germicidin	Inhibitor of germination	Germicidin (100) * S. scabiei* 87–22	[61]
30	SCAB_RS39275-39320 [SCAB_82111-NA]	14 262	Terpene	Cryptic	Unknown	None	na
31 a	SCAB_RS40100-40220 [SCAB_83841–84101]	94 968	Type 1 PKS	Concanamycins	Cytotoxic (antifungal, antineoplastic, anti- protozoal and antiviral)	Concanamycin A (89) * S. neyagawaensis *	[51, 62]
31b	SCAB_RS40225-40290 [NA-84261]	13 911	Linaridin	Cryptic	Unknown	None	na
32	SCAB_RS40385-40435 [SCAB_84461–84561]	13 579	Siderophore	Cryptic	Unknown	None	na
33 a	SCAB_RS40855-40900 [SCAB_85431–85521]	30 019	NRPS	Scabichelin	Iron uptake	Scabichelin (100) * S. scabiei* 87–22	[19]
33b	SCAB_RS40955-41010 [SCAB_85631–85741]	9584	Melanin	Melanin	Pigment	Melanin (57) * S. avermitilis *	BGC0000908
34	SCAB_RS41165-41255 [SCAB_86081–86261]	19 524	Terpene	Cryptic	Unknown	None	na

Over a third of the predicted BGCs (18 out of 46) contained genes involved in the production of natural products that have already been identified in *S. scabiei* or in other *Streptomyces* species (known BGCs) (Table 1). Half of these (nine out of 18) belonged to the so-called core metabolome [38] i.e. BGCs involved in the biosynthesis of molecules produced by almost all *Streptomyces* species including 2-methylisoborneol (BGC#5), isorenieratene (BGC#6 a), hopene (BGC#9), geosmin (BGC#12), the WhiE spore pigment (BGC#17), desferrioxamines (BGC#21), melanins (BGC#22, BGC#33b), and ectoine (BGC#24). The remaining nine BGCs of known metabolites were classified into three different functional categories, namely, (i) plant-associated molecules (thaxtomins, coronafacoyl phytotoxins, concanamycins, and rotihibins), (ii) siderophores (pyochelin, scabichelin and turgichelin, in addition to desferrioxamines), and (iii) antimicrobials (informatipeptin, bottromycins, and germicidins). Note that concanamycins also exhibit antiviral [39] and antifungal [40] activities due to their capacity to inhibit the V-type H^+^ ATPase [41] and therefore could also have been included into the ‘antimicrobials’ functional category.

The remaining 28 BGCs are considered as ‘cryptic’ or ‘orphan’, i.e. either their product is a yet undiscovered natural product (unknown unknowns) or is a known compound but the genetic material responsible for its synthesis is still unknown (unknown knowns) [42]. The compounds associated with these BGCs belonged to different natural products categories, i.e. five terpenes (BGC#6b, #14, #26, #30, #34), four NRPSs (BGC#1b, #11 a, #11b, #25), four PKSs (BGC#23 a, #23d, #27 a, #29 a), three siderophores (BGC#10, #15, #32), three lanthipeptides (BGC#4, #16b, #19), two bacteriocins (BGC#7b, #13), two butyrolactones (BGC#8, #23b), one LAP (BGC#23 c), one indole (BGC#27b), one linaridin (BGC#31b), one betalactone (BGC#2), and one hybrid PKS-NRPS BGC (BGC#18) (Table 1).

### Specialized metabolite production upon sensing cellobiose and cellotriose

**Fig. 1.**
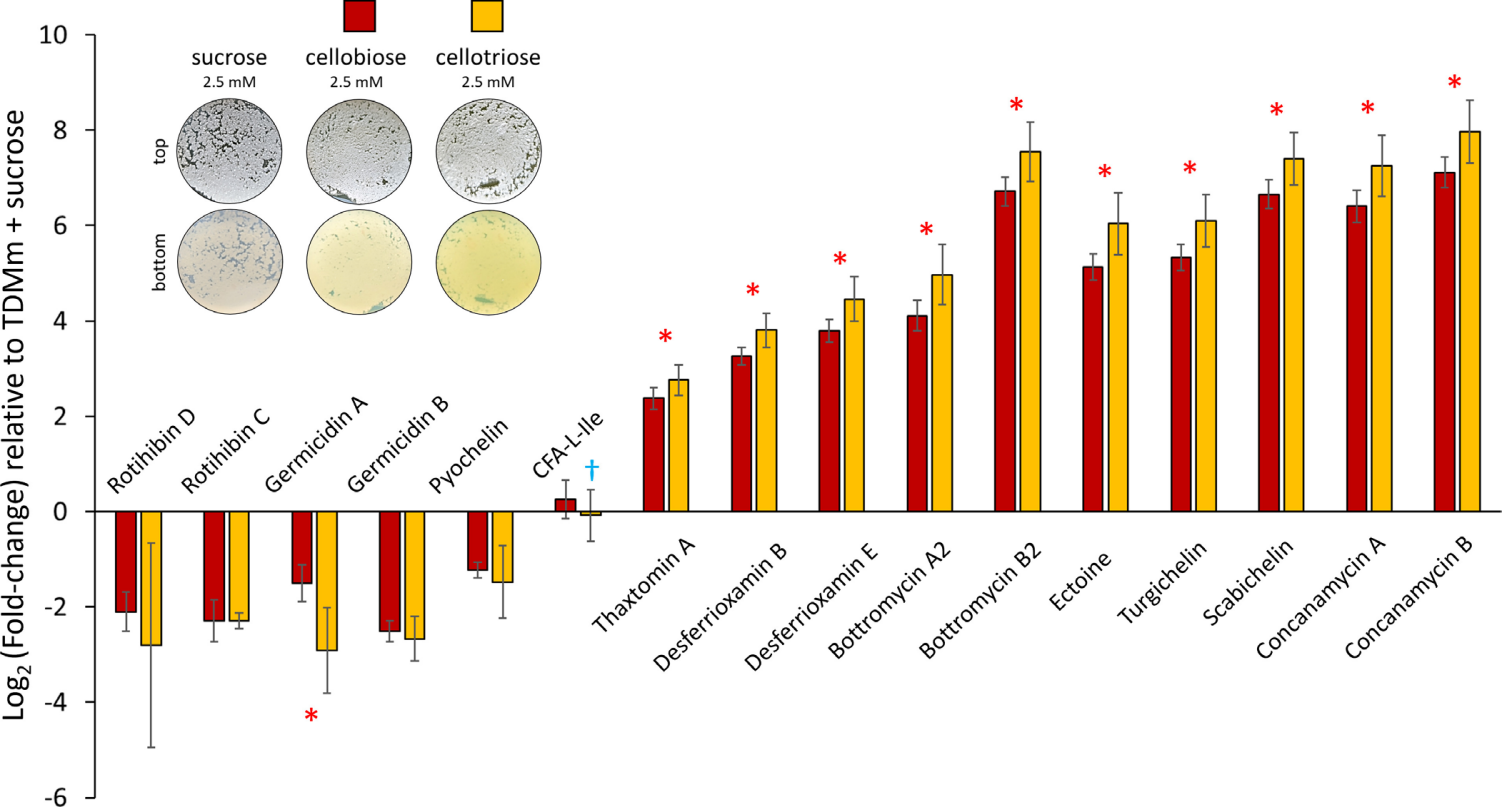
Log_2_ fold-change for production of the known specialized metabolites of *S *. *scabiei* 87–22 grown induced by cello-oligosaccharides. Production levels were assessed in three culture conditions (top left panel): TDM+maltose 0.5 % supplemented with (i) 2.5 mM of sucrose (control condition), (ii) 2.5 mM cellobiose, or (iii) 2.5 mM cellotriose. Bar plots display the Log_2_ fold-change of the mean of the normalized area under the curve of ion peaks detected in either cellobiose (red bars) or cellotriose (orange bars) culture conditions, compared to the mean of the normalized area under the curve of the same ion peak detected in the control ‘non-pathogenetic’ sucrose conditions. The error bars display the standard deviation observed between three biological replicates (each with two to three technical replicates). Note that comparison between cellobiose and cellotriose conditions against the sucrose condition were all significantly different (*P*-values<0.05, bilateral *t*-test) except for CFA-l-Ile in cellotriose (†). *, indicates metabolites whose production was statistically different between cellobiose and cellotriose (*P*-values<0.05).

The effect of cello-oligosaccharides cellobiose and cellotriose on the induction of the specialized metabolism of *S. scabiei* 87–22 was assessed by targeted liquid chromatography-multiple reaction monitoring MS (LC-MRM-MS). Fig. 1 shows the Log_2_(Fold-change) (LFC (cello-oligosaccaride/sucrose)) of the production of known metabolites of *S. scabiei* 87–22 when cultured with the environmental virulence elicitors cellobiose or cellotriose compared to the non-inducing condition (TDMm +sucrose). Expectedly, the level of thaxtomin A was significantly higher in TDMm +cellobiose and in TDMm +cellotriose than in TDMm +sucrose (LFC (cellobiose/sucrose)=2.4 and LFC (cellotriose/sucrose)=2.8). The greatest production of thaxtomin A appeared to occur in the presence of cellotriose compared to cellobiose, confirming earlier results in the closely related species *S. turgidiscabies* and *S. acidiscabies* suggesting that the trisaccharide has a higher triggering effect on thaxtomin phytotoxin biosynthesis [25]. Concanamycins A and B followed the same production pattern with stronger induction rates: on average 6.8- and 7.7-LFC increases in metabolite levels were observed in TDMm +cellobiose and in TDMm +cellotriose, respectively (Fig. 1). In contrast, the production of N-coronafacoyl-l-isoleucine (CFA-l-Ile) was not strikingly influenced by cello-oligosaccharides compared to other plant-associated metabolites (Fig. 1). Finally, both rotihibins (C and D), recently identified as a novel category of plant growth affecting compounds produced by *S. scabiei* [17], were underproduced with decreases of about 2.3-LFC following cellobiose and cellotriose supply (Fig. 1)

Regarding the production patterns of the siderophores produced by *S. scabiei* 87–22, both desferrioxamines (B and E) showed enhanced production when *S. scabiei* 87–22 was grown in the presence of cellobiose or cellotriose. Desferrioxamine E was the most overproduced of the two, especially in TDMm +cellotriose with an average of 4.5-LFC increase in its abundance levels (Fig. 1). Scabichelin and turgichelin – both synthesized by BGC#33 a – followed the same trend as desferrioxamines: production increases of about 6.1- and 6.9-LFC were observed upon addition of cellobiose and cellotriose, respectively. By contrast, pyochelin production was reduced of about 1.3-LFC by either of the cello-oligosaccharides (Fig. 1).

The production of bottromycins A2 and B2, as well as its other detected forms (D and E, data not shown), positively responded to the addition of cellobiose and cellotriose (Fig. 1). While there was on average a 5.9-LFC overproduction of these antimicrobial metabolites following the addition of cellobiose, cellotriose triggered about twice as much (6.8-LFC increase compared to the production in sucrose) the biosynthesis of bottromycins. The production of the osmoprotectant ectoine also positively responded to the presence of cellobiose and cellotriose, with 5.1- and 6-LFC overproduction, respectively (Fig. 1).

Out of all the analysed metabolites, one type of compounds had their relative abundance drastically decreased upon cello-oligosaccharide supply i.e. the germicidins. Germicidin A, the inhibitor of *Streptomyces* spore germination, showed a significant decrease in its production levels in both conditions containing cello-oligosaccharides – about 1.5-LFC in TDMm +cellobiose and 2.9-LFC in TDMm +cellotriose (Fig. 1). The production of germicidin B displayed the same pattern (Fig. 1).

### Transcriptional response of BGCs to cellobiose and cellotriose

### BGC of known metabolites under expression control of cello-oligosaccharides

**Fig. 2.**
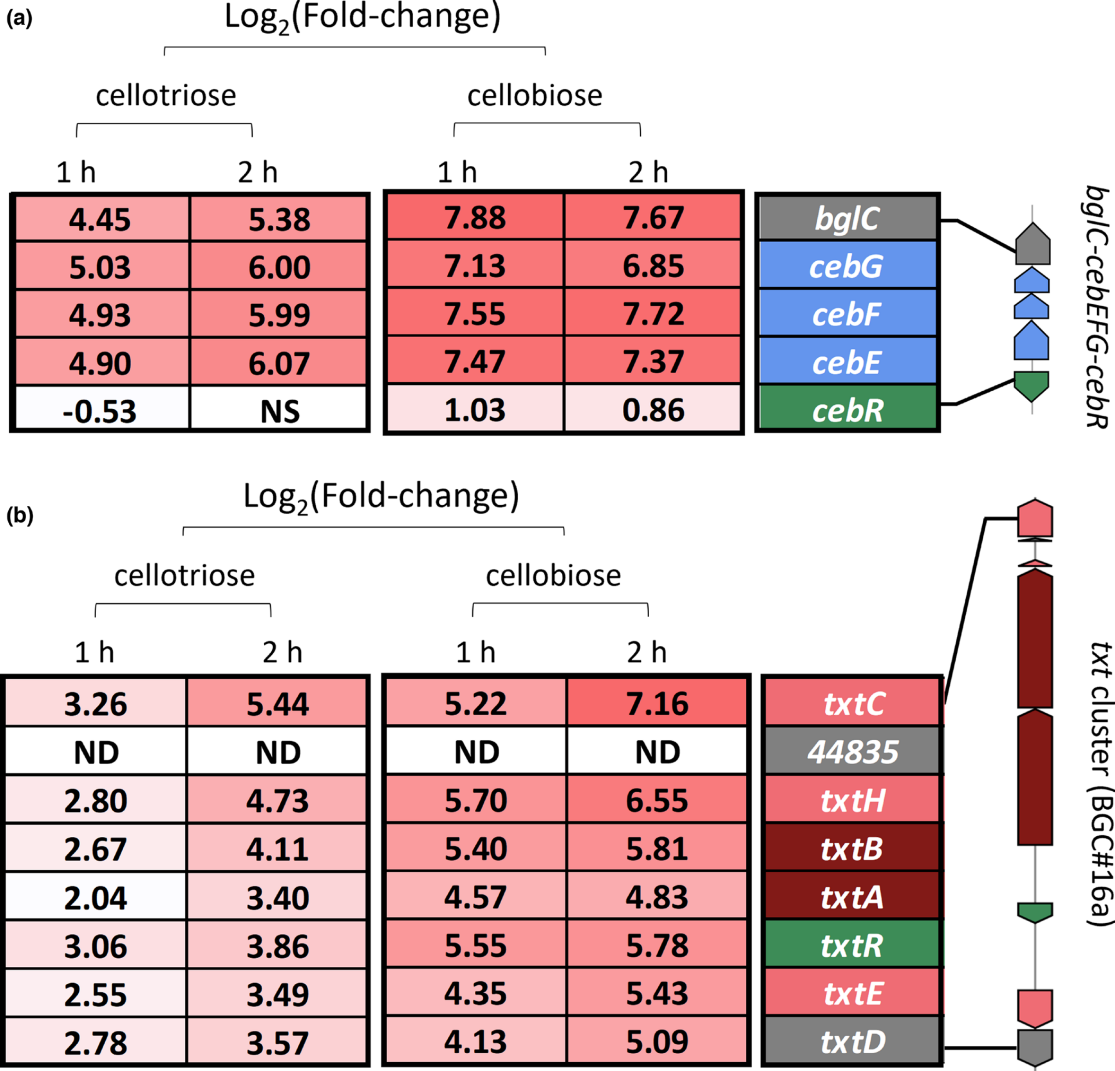
Log_2_(Fold-change) of transcription of genes involved in cello-oligosaccharide utilization (*cebR-cebEFG-bglC*, (**a**) and thaxtomin biosynthesis (*txt* cluster, (**b**) after addition of cello-oligosaccharides. Gene colour/function code: Green, regulatory genes; Blue, transport-related genes; Dark red, core biosynthetic genes; Pink: additional biosynthetic genes; Grey, others. nd, Not Detected.

Next to the metabolomic study described above, we also assessed which BGCs responded to the environmental triggers cellobiose and cellotriose at the transcriptional level by RNA-seq. For this, RNA samples were collected 1 and 2 h post-addition of cellobiose and cellotriose in order to monitor the instantaneous transcriptional effect of the triggers of the pathogenic lifestyle (see Methods for details). The expression response of genes encoding the cello-oligosaccharide ABC-transporter CebEFG and the expression of the gene encoding the beta-glucosidase BglC for subsequent hydrolysis into glucose were used as ‘positive controls’ for cellobiose/cellotriose upregulated genes. As shown in Fig. 2(a), the expression of the *cebEFG* operon was drastically induced by both cellobiose and cellotriose with on average 7.3- and 6-Log_2_(Fold-change) (LFC), respectively. Similarly, *bglC* displayed 7.7- and 5.4-LFC 2 h post-addition of cellobiose and cellotriose, respectively (Fig. 2a). The thaxtomin core biosynthetic genes, *txtA* (*scab_31791*) and *txtB* (*scab_31781*) are also known to be triggered by both cellobiose and cellotriose [25] and therefore can be regarded as additional ‘positive controls’ for cellobiose/cellotriose upregulated genes. The best transcriptional activation response for *txtA* and *txtB* was observed in the cellobiose condition, i.e. 4.8- and 5.8-LFC upregulation 2 h post-induction for *txtA* and *txtB*, respectively (Table 2, Fig. 2b). Cellotriose was similarly able to activate the expression of both genes, with the strongest fold-change also observed at 2 h post-induction, i.e. 3.3- and 4.1-LFC upregulation for *txtA* and *txtB*, respectively. Analysis of the expression patterns of the other *txt* genes revealed that the whole BGC positively responds to both elicitors, *txtC* displaying the strongest response in the cellobiose condition, with a 7.2-LFC increase at 2 h post-induction (Fig. 2b). Overall, the results obtained for the *cebEFG-bglC* operon and the *txt* cluster demonstrate that our experimental set up is appropriate to assess the transcriptional response of *S. scabiei* 87–22 upon sensing the presence of the triggers of virulence.

**Table 2.** Transcriptional response of BGCs (core genes) upon cellobiose and cellotriose supply. Mean Log_2_ fold-change of up and down regulated BGCs was highlighted in green (lime green below 2.9 log_2_ FC and jade green from 3.0 log_2_ FC and above) and pink, respectively.

BGC	Specialized metabolite	Locus tag (old locus tag)	Log_2_(Fold-change)					
			Cellobiose			Cellotriose		
			1h	2h	Mean	1h	2h	Mean
1a	**Pyochelin**	SCAB_RS00635 (SCAB_1411)	2.0±0.3	1.1±0.3	1.4	1.3±0.3	ns	1
		SCAB_RS00665 (SCAB_1471)	1.8±0.3	1.0±0.3		1.4±0.3	0.8±0.3	
		SCAB_RS00670 (SCAB_1481)	0.8±0.2	ns		0.6±0.2	ns	
1b	**Unknown**	SCAB_RS00695 (SCAB_1531)	ns	2.1±1.1	2.2	2.5±1.1	2.5±1.1	2.4
		SCAB_RS00700 (SCAB_1551)	2.4±0.5	1.8±0.5		2.5±0.5	2.1±0.5	
2	**Unknown**	SCAB_RS00900 (SCAB_1671)	ns	ns	–	ns	3.5±1.7	2.9
		SCAB_RS00915 (SCAB_2041)	ns	ns		1.8±0.9	ns	
3	**Rothibins**	SCAB_RS01525	−0.5±0.2	−0.4±0.2	−0.5	ns	−0.8±0.2	−0.8
4	**Unknown**	SCAB_RS01675 (SCAB_3621)	0.9±0.4	ns	0.9	ns	ns	–
5	**2-MIB**	SCAB_RS02330 (SCAB_5041)	1.5±0.4	1.6±0.4	1.6	1.7±0.4	1.6±0.4	1.7
6a	**Isorenieratene**	SCAB_RS02515 (SCAB_5441)	ns	−0.7±0.3	−0.7	ns		0.7
		SCAB_RS02535 (SCAB_5441)	ns	ns		ns	0.7±0.2	
6b	**Unknown**	–	ns	ns	–	ns	ns	–
7a	**Informatipeptin**	SCAB_RS04055 (SCAB_8611)	ns	ns	–	ns	ns	–
7b	**Unknown**	SCAB_RS04085 (SCAB_8681)	ns	2.2±0.9	2.2	2.0±0.9	2.8±0.9	2.5
8	**Unknown**	SCAB_RS05740 (SCAB_12081)	ns		–	ns	ns	–
9	**Hopene**	SCAB_RS06160 (SCAB_8681)	−0.6±0.3	−1.0±0.3	−0.8	ns	ns	0.9
		SCAB_RS06175 (SCAB_12991)	ns	ns		0.9±0.4	1.0±0.4	
10	**Unknown**	SCAB_RS08695 (SCAB_18391)	−0.5±0.2	ns	−0.5	−0.4±0.2	−0.6±0.2	−0.7
		SCAB_RS08700 (SCAB_18401)	ns	ns		ns	−1.4±0.6	
11 a	**Unknown**	SCAB_RS09315 (SCAB_19691)	ns	ns	–	ns	ns	–
11b	**Unknown**	–	–	–	–	–	–	–
12	**Geosmin**	SCAB_RS09510 (SCAB_20121)	−1.2±0.3	−1.0±0.3	−1.1	−0.7±0.3	−1.0±0.3	−0.8
13	**Unknown**	SCAB_RS09810 (SCAB_20761)	1.6±0.2	1.9±0.2	1.7	1.5±0.2	1.9±0.2	1.7
14	**Unknown**	SCAB_RS10960 (SCAB_23181)	1.0±0.2	1.7±0.2	1.4	0.6±0.2	0.9±0.2	0.8
15	**Unknown**	SCAB_RS11650 (SCAB_24681)	1.6±0.3	1.4±0.3	1.5	1.4±0.3	1.5±0.3	1.4
16 a	**Thaxtomins**	SCAB_RS15080 (SCAB_24681)	5.4±0.3	5.8±0.3	5.2	2.7±0.3	4.1±0.3	3.3
		SCAB_RS15085 (SCAB_31791)	4.6±0.3	4.8±0.3		2.0±0.3	3.4±0.3	
16b	**Unknown**	SCAB_RS15175 (SCAB_32031)	0.5±0.2	ns	0.6	ns		0.7
		SCAB_RS15180 (SCAB_32041)	ns	ns		ns	1.0±0.4	
		SCAB_RS15185 (SCAB_32051)	ns	0.6±0.2		0.6±0.2	0.5±0.2	
17	**Spore pigment**	SCAB_RS20600 (SCAB_43301)	ns	ns	–	ns	ns	–
		SCAB_RS20605 (SCAB_43311)	ns	ns		ns	ns	
18	**Unknown**	SCAB_RS20905 (SCAB_43961)	−0.5±0.2	−0.5±0.2	−0.5	ns	−0.9±0.2	−0.9
19	**Unknown**	SCAB_RS22675 (SCAB_47641)	0.7±0.3	ns	0.7	ns	ns	–
20	**Bottromycins**	SCAB_RS27005 (SCAB_56611)	0.9±0.5	ns	−0.4	1.4±0.4	ns	−0.5
		SCAB_RS27040 (SCAB_56691)	−1.0±0.4	−1.7±0.4			−1.7±0.4	
21	**Desferrioxamines**	SCAB_RS27660 (SCAB_57921)	2.0±0.4	5.1±0.4	4.3	2.5±0.4	4.8±0.4	4.1
22	**Melanin**	SCAB_RS28265 (SCAB_59231)	ns	ns	–	2.4±0.9	3.2±0.9	2.8
23 a	**Unknown**	SCAB_RS30055 (SCAB_62941)	ns	1.6±0.7	1.6	ns	ns	–
23b,c,d	**Unknown**	SCAB_RS30160 (SCAB_63151)	ns	ns	–	0.8±0.4	0.8±0.4	0.8
24	**Ectoine**	SCAB_RS33840 (SCAB_70721)	ns	−0.6±0.3	−0.6	−0.9±0.3	−1.9±0.3	−1.3
25	**Unknown**	SCAB_RS34930 (SCAB_72991)	1.0±0.3	1.4±0.3	1.2	ns	0.8±0.3	0.8
26	**Unknown**	SCAB_RS35290 (SCAB_73741)	−0.4±0.2	−0.7±0.2	−0.5	−0.4±0.2	−0.7±0.2	−0.6
27 a	**Unknown**	SCAB_RS37805 (SCAB_78961)	ns	ns	–	ns	ns	–
27b	**Unknown**	SCAB_RS37920 (SCAB_79221)	0.9±0.2	0.9±0.2	0.9	0.5±0.2	0.6±0.2	0.6
28	**Coronafacoyl phytotoxins**	SCAB_RS38130 (SCAB_79651)	3.8±0.3	2.7±0.3	3.9	3.7±0.3	3.7±0.3	4.4
		SCAB_RS38135 (SCAB_79661)	4.5±0.3	4.0±0.3		4.5±0.3	5.1±0.3	
29 a	**Unknown**	–	–	–	–	–	–	–
29b	**Germicidins**	SCAB_RS38390 (SCAB_80171)	ns	ns	–	ns	ns	–
30	**Unknown**	SCAB_RS39305 (SCAB_82161)	ns	ns	–	ns	ns	–
31 a	**Concanamycins**	SCAB_RS40110 (SCAB_83871)	ns	1.0±0.5	1.4	ns	ns	1.6
		SCAB_RS40115 (SCAB_83891)	1.3±0.4	1.2±0.4		0.9±0.5	1.8±0.4	
		SCAB_RS40120 (SCAB_83901)	ns	ns		ns	1.5±0.6	
		SCAB_RS40125 (SCAB_83911)	1.6±0.5	1.3±0.6		ns	1.4±0.5	
		SCAB_RS40130 (SCAB_83921)	ns	1.7±0.7		ns	2.2±0.8	
		SCAB_RS40135 (SCAB_83931)	ns	ns		ns	3.0±1.2	
31b	**Unknown**	SCAB_RS40250 (SCAB_84181)	ns	ns	–	ns	ns	–
32	**Unknown**	SCAB_RS40405 (SCAB_84501)	3.3±0.4	5.0±0.4	4.2	2.8±0.4	4.1±0.4	3.4
		SCAB_RS40410 (SCAB_84511)	2.7±0.5	4.6±0.5		2.3±0.5	3.6±0.5	
33 a	**Scabichelin / Turgichelin**	SCAB_RS40875 (SCAB_85471)	3.2±0.2	4.0±0.2	3.7	2.8±0.2	3.5±0.2	3.2
33b	**Melanin**	SCAB_RS40985 (SCAB_85691)	ns	ns	–	ns	ns	–
34	**Unknown**	SCAB_RS41210 (NA)	ns	ns	–	ns	ns	–

**Fig. 3.**
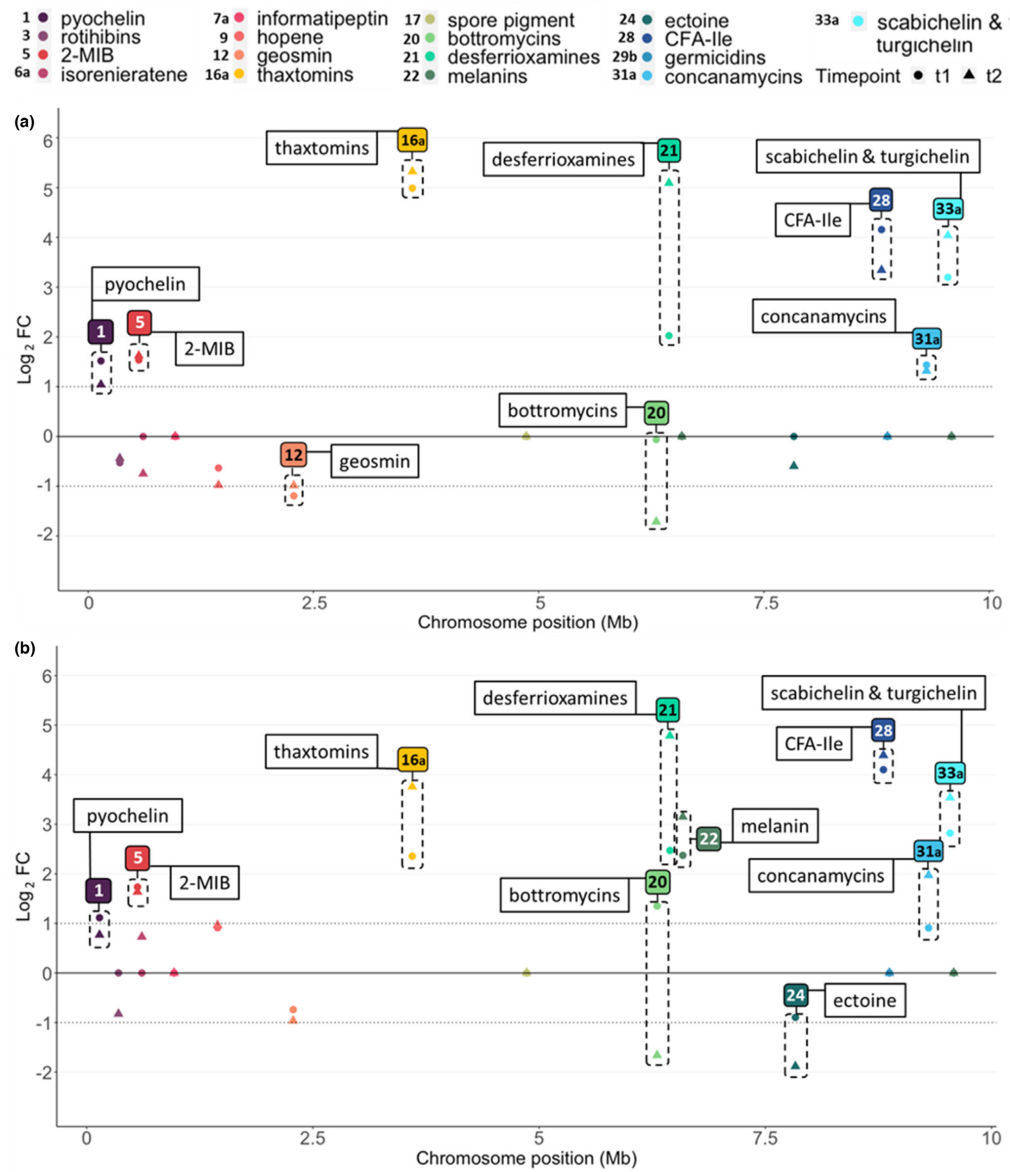
Expression response of core genes of the known BGCs in presence of cellobiose (**a**) and cellotriose (**b**). The y-axis presents the Log_2_ of the expression fold-change (FC) of core biosynthetic genes upon addition of cellobiose (**a**) and cellotriose (**b**). Circles and triangles indicate Log_2_FC measured at 1 and 2 h post-addition of cello-oligosaccharides, respectively. The x-axis presents the position of BGCs on the chromosome. Only data with lfcSE <0.05 (standard error of the log_2_FC estimate) are displayed (BGCs not meeting this criterion have been set to 0). BGCs with a fold-change above or below the threshold −1 > Log_2_FC>1 (at least one time point) are highlighted by a dotted frame.

From all expression data, we first focused on the genes belonging to the 18 BGCs involved in the production of known specialized metabolites of *S. scabiei* 87–22 (Table 2, Fig. 3). We only considered the expression data of the core biosynthetic gene(s) of each BGC (Table S2) in order to have the best possible correlation between the transcriptomic data and the metabolomic study described earlier. The transcriptional response of the core biosynthetic genes of these ‘known’ BGCs upon supply of cellobiose and cellotriose is displayed in Fig. 3(a, b), respectively, and fold-change values for the core biosynthetic genes of each BGC are displayed in Table 2. The correlation between data deduced from the metabolomic and transcriptomic approaches are displayed in Fig. S1.

Next to BGC#16 a for thaxtomin production, the other BGCs involved in the production of plant-associated molecules also positively responded to cello-oligosaccharides. As mentioned following the metabolomic (Fig. 1), the production of concanamycins was highly responsive to both cello-oligosaccharides. The transcriptomic study confirms this, as the expression of the core biosynthetic genes of BGC#31 a, namely *conABCDEF*, showed an average of 1.4- and 1.6-LFC upregulation in cellobiose and in cellotriose, respectively. Genes of BGC#28 responsible for CFA-l-Ile production also showed a very strong increase in expression in response to both cello-oligosaccharides (an average of 3.9- and 4.4-LFC upregulation for cellobiose and cellotriose, respectively, (Table 2, Fig. 3)), which contrasts with the weak production changes deduced from the metabolomic approach (Figs 1 and S1). Finally, the analysis of the biosynthetic genes of rotihibins (BGC#3) revealed that the expression of core genes of this cluster was repressed by both cellobiose and cellotriose though at LFC of −0.5 and −0.8, respectively, thus slightly below the fixed threshold of LFC of −1 for significant changes (Table 2, Fig. 3). The results of our metabolomic analysis revealed a much stronger reduced production of rotihibins (Fig. 1).

Regarding the biosynthetic genes of desferrioxamines (BGC#21), scabichelin and turgichelin (BGC#33 a) siderophores, their expression was also greatly influenced by both saccharides. Transcription of the core biosynthetic gene responsible for desferrioxamines biosynthesis displayed its greatest response 2 h post-induction for both elicitors, *i.e*. 5.1- and 4.8-LFC upregulation for cellobiose and cellotriose, respectively. The biosynthetic gene of BGC#33 a involved in scabichelin and turgichelin production was similarly positively affected by cellobiose and cellotriose with an average of 3.7- and 3.2-LFC upregulation, respectively (Table 2, Fig. 3). The metabolomic data suggested that pyochelin is the only siderophore whose production was repressed and not activated by cello-oligosaccharides (Fig. 1). However, the expression of pyochelin biosynthetic genes (BGC#1 a) instead revealed a small but significant expression increases of 1.4- and 1.0-LFC upon addition of cellobiose and cellotriose, respectively (Table 2, Fig. 3).

Regarding the BGCs responsible for the production of the antimicrobial compounds the transcription of BGC#20 (for bottromycins) displayed contradicting transcriptional responses (up-regulated or no change at 1 h post-induction and down-regulated at 2 h post-induction, Fig. 3), that overall are not in line with the remarkable overproduction observed via the comparative metabolomic approach (Fig. 1, see Discussion). BGC#7a, which shows about 60 % synteny to the antimicrobial lanthipeptide informatipeptin, had an expression pattern that was neither influenced by cellobiose nor cellotriose (Table 2, Fig. 3). Finally, the LFC for BGC#29b associated with germicidins was not lower than the threshold probability value of *P*≤0.05. The lack of significant expression change did not allow us to correlate transcriptomic data with the marked decrease in germicidin production deduced from the metabolomic analysis (Fig. 1, see Discussion).

Regarding the nine BGCs which belong to the so-called core metabolome of *Streptomyces* species, the addition of cello-oligosaccharides only significantly influenced the expression of BGC#5 (2-methylisoborneol), BGC#12 (geosmin), and BGC#22 (melanin) (Fig. 3, Table 2). The effect of both inducers on the expression of the desferrioxamine BGC – which is part of the core metabolome – has already been discussed in the section associated with siderophore BGCs. Cellobiose and cellotriose both activated expression of BGC#5 at almost the same level – around 1.6-LFC, whereas only cellotriose had an impact on the expression of the biosynthetic genes of BGC#22 with an average of 2.8-LFC upregulation. Regarding the osmoprotectant ectoine (BGC#24 in Table 1), we observed a weak but significant reduced expression in cellobiose which does not correlate with the overproduction measured via the metabolomic approach (Figs 1 and S1).

### Cryptic BGCs transcriptionally activated by cello-oligosaccharides

**Fig. 4.**
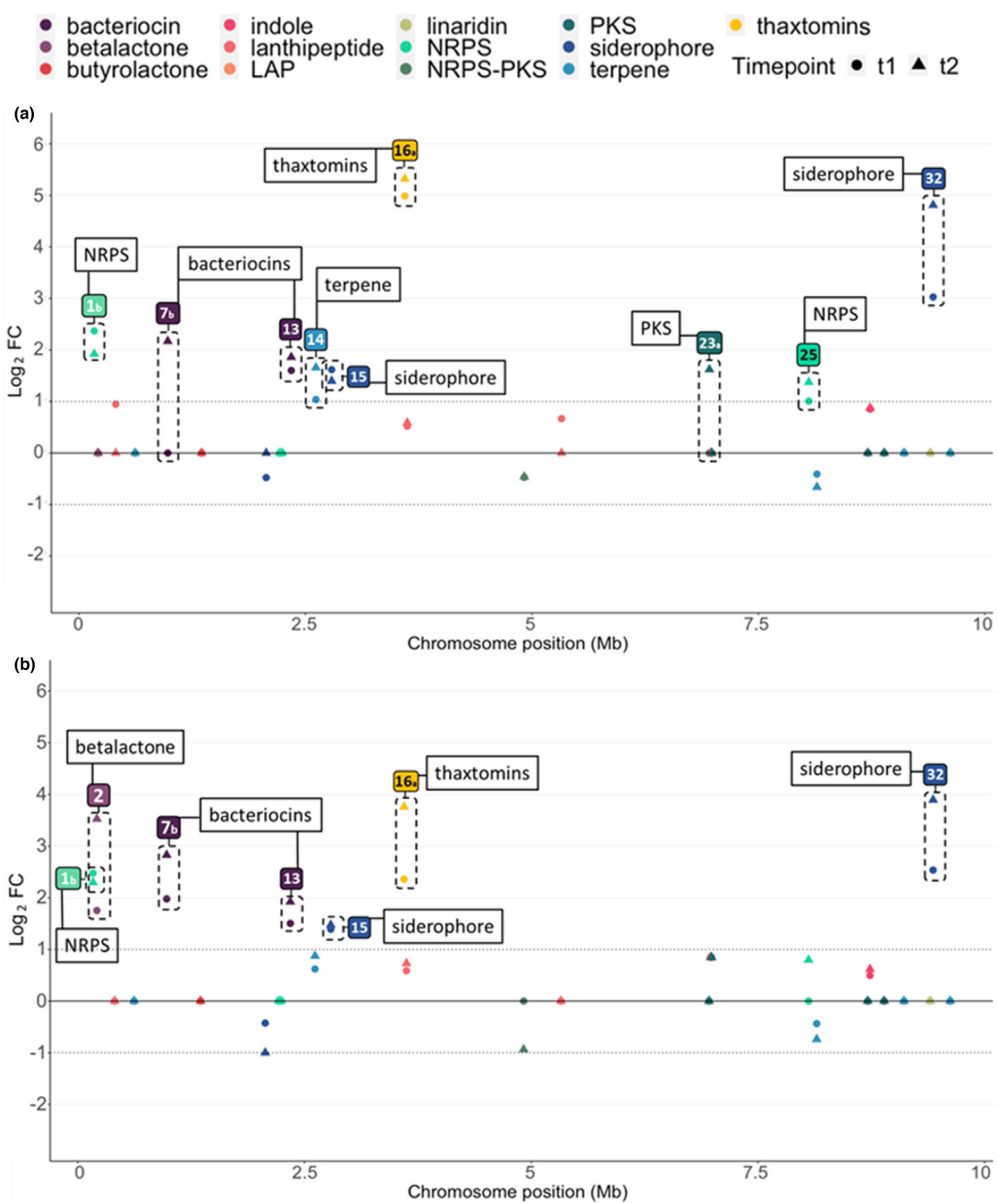
Expression response of core genes of the cryptic BGCs in presence of cellobiose (**a**) and cellotriose (**b**). The y-axis presents the Log_2_ of the expression fold-change (FC) of core biosynthetic genes upon addition of cellobiose (**a**) and cellotriose (**b**). Circles and triangles indicate Log_2_FC measured at 1 and 2 h post-addition of cello-oligosaccharides, respectively. The x-axis presents the position of BGCs on the chromosome. Only data with lfcSE<0.05 (standard error of the log_2_FC estimate) are displayed (BGCs not meeting this criterion have been set to 0). BGCs with a fold-change above or below the threshold −1 > Log_2_FC>1 (at least one time point) are highlighted by a dotted frame.

Aside from the 18 BGCs involved in the production of known metabolites, we also assessed the effect of each cello-oligosaccharide on the expression level of the 28 cryptic or orphan BGCs deduced through genome mining (Tables 1 and 2). As shown in Fig. 4, the expression of nine cryptic BGCs was influenced by cellobiose or cellotriose. Both carbohydrates significantly increased the transcription of five BGCs, namely BGC#1b (NRPS), BGC#7b (bacteriocin), BGC#13 (bacteriocin), BGC#15 (siderophore), and BGC#32 (siderophore) (Table 2). The highest transcriptional response was observed for the genes of BGC#32 involved in the synthesis of a siderophore metabolite, which were induced up to 5-LFC by cellobiose and 4.1-LFC by cellotriose (Table 2, Fig. 4). The transcription of another BGC coding for an unknown siderophore (BGC#15) was also increased to about 1.5-LFC by both carbon sources (Table 2, Fig. 4). Interestingly, both bacteriocin types of metabolites, i.e. BGC#7b and BGC#13, were upregulated by both cello-oligosaccharides. BGC#13 was equally upregulated by both sugars (about 1.7-LFC), whereas the transcriptional response of BGC#7b was induced more by cellotriose (2.8-LFC) compared to cellobiose (2.2-LFC). The presence of cellobiose and cellotriose also positively influenced the transcription of two unknown NRPS BGCs (BGC#1b and BGC#25) with an average 2.3-LFC change for BGC#1b and around 1.2-LFC change for BGC#25 (Table 2, Fig. 4).

Four other BGCs had their expression upregulated by only one of the two tested cello-oligosaccharides. Cellobiose induced the expression of BGC#14 (terpene), BGC#23 a (PKS), and BGC#25 (NRPS) (Fig. 4a), whereas cellotriose positively impacted the expression of BGC#2 (betalactone) (Fig. 4b). Importantly, the expression of the core genes of five of the 28 cryptic BGCs was not significantly influenced by cellobiose or cellotriose. The expression of the other 14 cryptic BGCs remained silent under the condition tested.

### Effect of *cebR* deletion on the expression of cello-oligosaccharide-dependent BGCs

**Fig. 5.**
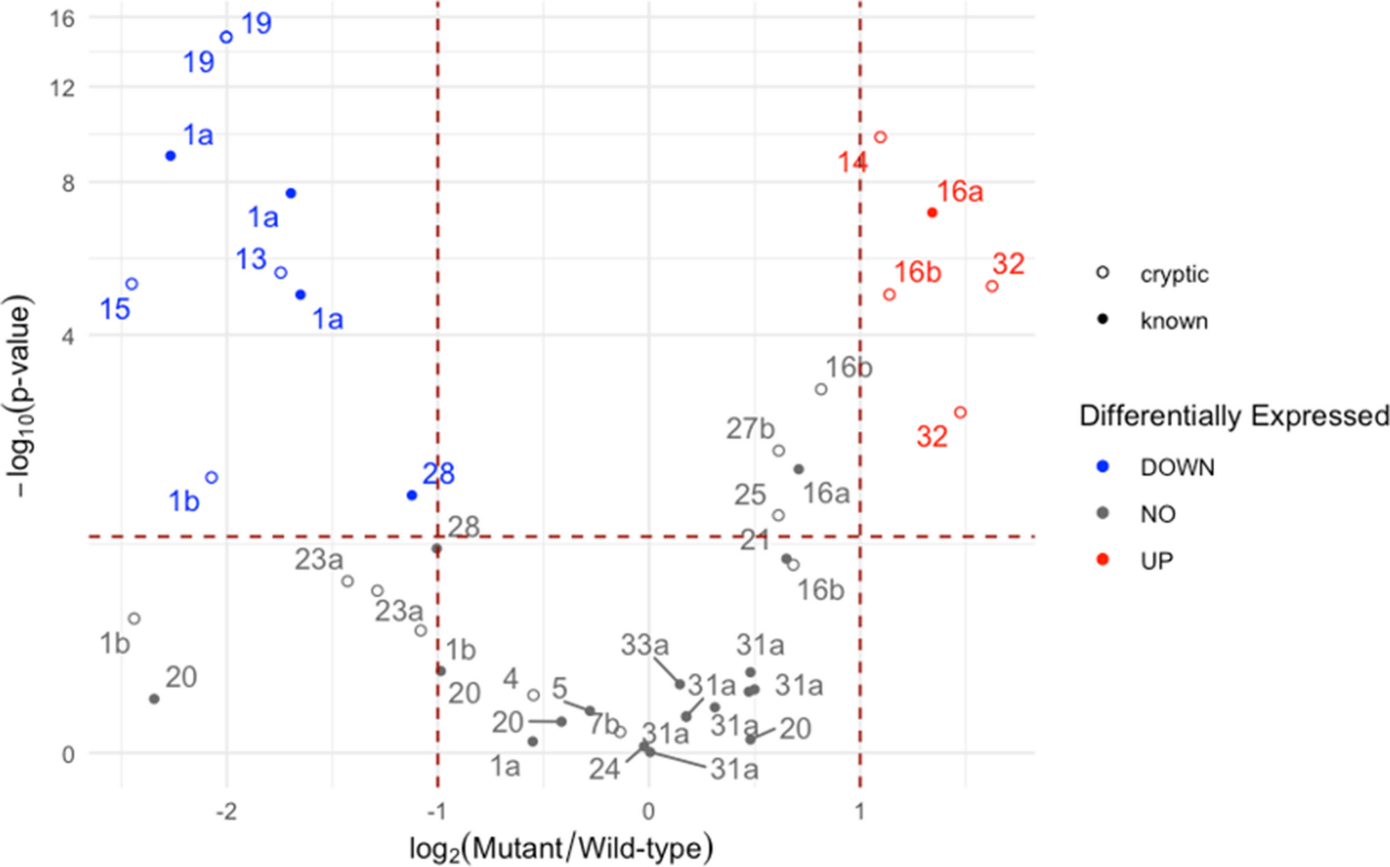
Volcano plot displaying differentially expressed core BGC genes between the *S. scabiei* wild-type strain and the ∆*cebR* mutant. Genes belonging to cryptic BGCs are represented by an empty circle, and those from known BGCs by a full one. Colours indicate the differential expression of each core gene in the ∆*cebR* mutant strain relative to the WT: upregulated (red), downregulated (blue), no significant change (grey). The x-axis displays the Log_2_ fold-change (FC) between the mutant and the WT, while the y-axis corresponds to the -Log_10_ (p-value). Significant expression changes were defined as having a p-value<0.05 and a Log_2_FC above or below the given threshold, 1 and −1 respectively (−1 > Log_2_FC>1), these limits are represented by dotted red lines on the plot.

The *txt* cluster responsible for thaxtomin production was previously reported to be under direct control of the cellulose utilization repressor CebR [22]. Two CebR-binding sites have been discovered within the *txt* cluster which allows the CebR repressor to switch off the expression of the thaxtomin pathway-specific activator TxtR, in turn resulting in the transcriptional repression of the whole thaxtomin BGC. Binding of cellobiose and/or cellotriose to CebR unlocks the system which allows thaxtomin production. According to our transcriptomic analysis, a total of 16 BGCs had their expression increased by the addition of either cellobiose and/or cellotriose (Table 2) suggesting a possible role of CebR as direct transcriptional repressor of other gene clusters of *S. scabiei *. A transcriptome analysis was thus performed in order to assess which BGCs, beyond the *txt* cluster, also have their expression under control of CebR. For this, *S. scabiei* 87–22 (wild-type) and its *cebR* null mutant (Δ*cebR*) were cultured in ISP2 liquid medium, and RNA samples were collected 3 h after culture inoculation with fresh mycelium. The volcano plot in Fig. 5 shows the relative expression of genes that were determined to be ‘core biosynthetic genes’ of the different BGCs (Table S2). As can be seen in this plot (Fig. 5), only one ‘known’ BGC showed an increased expression in the ∆*cebR* mutant, which unsurprisingly corresponds to the thaxtomin biosynthetic cluster (BGC#16 a). However, three additional cryptic clusters saw their core genes’ expression increased, namely, BGC#14, #16b, and #32 coding for terpene, lanthipeptide, and siderophore specialized metabolites, respectively. In the case of BGC#16b, we could also observe that only one out of its three core genes fall into the upregulated category (‘UP’ at Fig. 5). Interestingly, BGC#32 also responded positively to cellobiose and cellotriose (Table 2), and BGC#14 also showed upregulation upon cellobiose supply (Table 2). However, none of the genes from these cryptic BGCs have been predicted to contain a CebR-binding site (*cbs*) in their upstream region, meaning it is unlikely that their expression would be directly regulated by CebR (see Discussion).

Based again on core gene differential expression, there is a total of six downregulated BGCs, among which two known clusters (BGC#1 a and #28), corresponding to the pyochelin and CFA-l-Ile biosynthetic clusters. However, these two known BGCs showed upregulation upon cellobiose and/or cellotriose supply (Table 2) suggesting a possible absence of correlation between the response to cello-oligosaccharides and the inactivation of the DNA-binding ability of CebR. The four cryptic BGCs that were downregulated correspond to types NRPS (BGC#1b), bacteriocin (BGC#13), siderophore (BGC#15), and lanthipeptide (BGC#19).

## Discussion

### Conclusions on metabolites that respond to cello-oligosaccharides

Of all the different ways to determine what makes an organism excel in a lifestyle or in an environmental niche, generating mutants and assessing their phenotypic repercussions is a straightforward approach. However, this approach can sometimes lead to erroneous or questionable conclusions for various reasons, such as gene-function redundancy or genetic compensation mechanisms that could lead to phenotypes that understate the importance of a gene. In studies on CS disease, finding what is essential for the virulence of *S. scabiei* and related pathogenic species is thus subjected to these constraints linked to the use of reverse genetics. For instance, inactivation of *scab_1471* in *S. scabiei* resulted in a mutant strain unable to produce pyochelin but showing no sign of reduced virulence, indicating that this siderophore is either not essential for pathogenicity, or that its absence is rescued by other siderophore(s) produced by *S. scabiei* [20]. Also, the interference with thaxtomin production caused by the inactivation of the cellobiose and cellotriose beta-glucosidase BglC [28] revealed a genetic compensation phenomenon that awakened the expression of alternative beta-glucosidases allowing *S. scabiei* to maintain the capacity to use cello-oligosaccharides [43]. The experimental set-ups can also sometimes be suboptimal – such as inappropriate host and/or culture conditions – to observe the real impact of a mutation and therefore to conclude on the role of a gene product in a biological process. This is for example the case of CFA-l-Ile as gene inactivation in *S. scabiei* showed reduced tissue hypertrophy on potato tuber slices, but the impact of the mutant *in vivo* has only been assessed on tobacco and not on its natural hosts which questions the relevance of this molecule in the colonization process [44]. Apart from the results on the mutants involved in the biosynthesis of thaxtomins [5, 22, 23, 28], it is thus sometimes difficult to draw conclusions on the importance of a BGC in contributing to the capacity of *S. scabiei* to colonize and infect root and tuber plants.

**Fig. 6.**
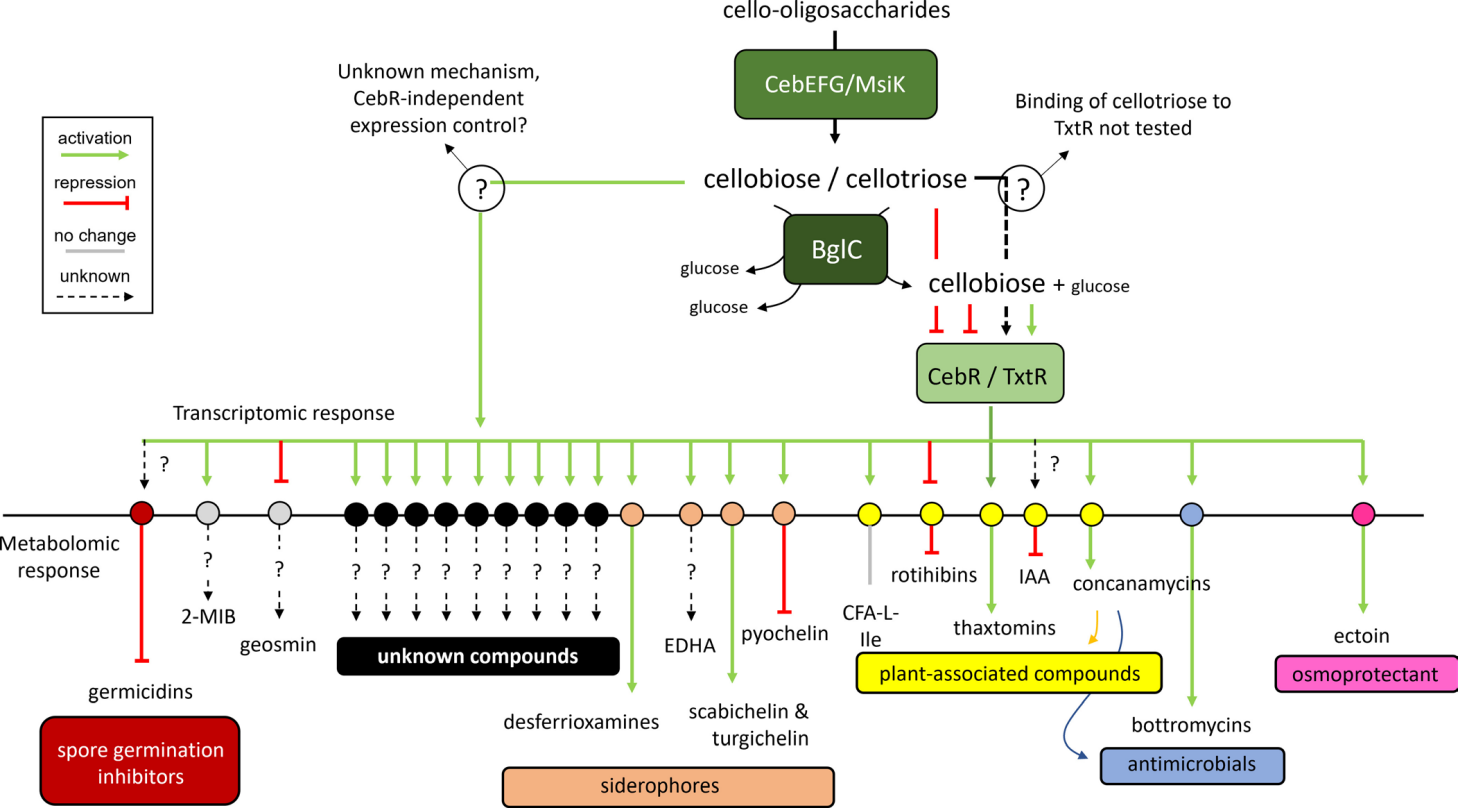
Transcriptomic and metabolomic response of the specialized metabolism of *S *. *scabiei* 87–22 in response to cello-oligosaccharides.

For the above-mentioned reasons, we chose approaches alternative to reverse genetics, i.e., comparative transcriptomic and metabolomic analyses, to determine which part of the specialized metabolism of *S. scabiei* would be dedicated to host infection (summarized in Fig. 6). Both approaches assume that a large proportion of the molecules/genes required for host colonization will respond to the same elicitors and therefore will display production or expression patterns that are synchronized with the main virulence determinants, in this case, the thaxtomin phytotoxins. The results of the metabolomic approach provided a clear picture of the specialized metabolites of *S. scabiei* that have their production specifically modulated by cello-oligosaccharides (Figs 1 and 6). The cello-oligosaccharide-dependent known metabolites of the virulome of *S. scabiei* include: (i) plant-associated metabolites, namely thaxtomins and concanamycins phytotoxins (and to a lesser extent CFA-l-Ile), (ii) desferrioxamines, scabichelin and turgichelin siderophores, (iii) the bottromycin antimicrobials, and (iv) the osmoprotectant ectoine. Importantly, germicidins, that are autoregulatory inhibitors of *Streptomyces* spore germination, are metabolites that had their production sensibly reduced. Inhibition of germicidin production following the perception of cellotriose that would emanate from the plant cell wall could be regarded as the first ‘green light’ to allow the onset of the pathogenic lifestyle of *S. scabiei *. Moreover, the production of the plant growth regulators rotihibins was drastically reduced after addition of cellobiose and cellotriose. This response, opposite to the dynamics of thaxtomins, suggests that rotihibins, if they are part of the virulome of *S. scabiei *, would not act as phytotoxins. The presence of clusters homologous to BGC#3 in plant-helping streptomycetes, and the plant growth promoting effect observed at low doses suggest that rotihibins might be involved in another aspect of the plant-associated lifestyle, despite exhibiting phytotoxicity at higher doses [17].

Our work showed that there is also a strong positive biosynthetic response of desferrioxamines, scabichelin and turgichelin siderophores which are molecules usually produced upon sensing low iron concentrations. Moreover, a further survey of the literature revealed the existence of a seventh BGC associated with the production of siderophores in *S. scabiei *. This 11 kb cluster ranges from *scab_5231* to *scab_5311* and is associated with the production of ethylenediaminesuccinic acid hydroxyarginine (EDHA) [45]. Due to the limited knowledge on the molecular signature of the biosynthetic elements constituting this type of BGC, the genes associated with EDHA production have not been detected by antiSMASH and this BGC was therefore missed in our genome mining analysis, as well as in the one performed by Liu and colleagues [29]. Interestingly, the transcription of the whole EDHA BGC showed a strong positive response to the addition of both cello-oligosaccharides, with core biosynthetic genes displaying up to 4.65-LFC expression increases (Fig. S2). Pyochelin is thus the only siderophore for which we observed a significant decreased production upon sensing the elicitors of virulence (Figs 1 and 6). Siderophores and iron supply are essential for the onset of both metabolite production and sporulation in streptomycetes [46–48]. We have previously shown that when siderophore biosynthesis responds to signals other than the environmental iron concentration, it can have a strong impact on their developmental programme [49]. Such a synchronized production of phytotoxins and siderophores, though still an enigma regarding the molecular mechanism in place, makes sense in terms of host colonization. Iron is mandatory for most housekeeping functions yet free iron is not available within the host. The upregulation of two additional cryptic BGCs predicted to be involved in the biosynthesis of siderophores (see below) further emphasizes the essential role of iron acquisition during host colonization. Despite the lack of metabolomic data, the production of EDHA could possibly also be part of the virulome of *S. scabiei* responsive to cellobiose and cellotriose. Interestingly, Liu *et al*. 2021 reported the absence of scabichelin and turgichelin in OBA [19, 29], suggesting that other compounds present in this complex medium interfere with the elicitor role of cello-oligosaccharides. It has to be noted that production of turgichelin - together with scabichelin - by *S. scabiei* (BGC#33 a) is reported for the first time in this work. Among the other metabolites whose production differed with the metabolomic analysis performed on OBA [29], are the bottromycins. These metabolites were detected in OBA, although in very low quantities, while we showed that cello-oligosaccharides strongly induce their production. On the other hand, compounds that were reportedly produced to high levels in OBA – such as concanamycins, thaxtomins, and desferrioxamines – were also detected in large quantities upon cello-oligosaccharide addition.

Furthermore, Liu and colleagues [29] highlighted the abundance of CFA-l-Ile in OBA culture extracts (also shown in [44]). The presence of CFA-l-Ile in our culture extracts suggests an important role for this molecule in the virulome, even though the addition of cello-oligosaccharides only had a limited positive impact on CFA-l-Ile production (Fig. 1), while we observed a significant increase in expression via our transcriptomic analysis (Fig. 3, Table 2). Surprisingly, we previously reported in a proteomic study that the abundance of two proteins of the CFA-l-Ile biosynthetic pathway – SCAB79611 (Cfa2) and SCAB79671 (CFL) – significantly decreased upon cellobiose addition [50]. Altogether, these results suggest that the production levels and the expression response of CFA-l-Ile biosynthetic proteins/genes are highly sensitive to the chosen culture conditions which could explain the important differences between our experimental setup conducted here in minimal media and earlier studies.

Additional metabolites have been identified in a previous study [29] and some of these were found in all our culture conditions (Fig. S3). Production of aerugine and a form of the plant hormone auxin (Indole-3-acetic acid - IAA) was significantly reduced by a factor two to four (LFC −1 to −2) in TDMm +cellobiose and cellotriose, respectively (Fig. S3). Andrachcinidine, an alkaloid metabolite putatively involved in plant defence, tended to be induced by cellobiose but repressed by cellotriose. Further research is required to link these metabolites with their currently cryptic BGCs, except for IAA whose biosynthetic genes have previously been identified [51]. On the other hand, the presence of additional metabolites described by Liu and colleagues [29] was investigated, but none of them was found in our extracts most likely due to the use of different culture media. These metabolites were: mairine B, bisucaberin, dehydroxynocardamines, and 211 A decahydroquinoline. Informatipeptin – a bioactive compound associated with antimicrobial activity – most-likely produced by BGC#7 a could not be detected in any of our extracts.

### Cellobiose versus cellotriose as elicitors of virulence

Another question we wanted to address through this work is whether cellotriose can, equally to cellobiose, trigger the ‘virulome’ of *S. scabiei *. Indeed, most studies have been performed with cellobiose as elicitor – the product being much less expensive and available in larger quantities compared to cellotriose, although cellobiose has never been shown to emanate from living plants considering the cell wall-related action of thaxtomins. Instead, cellotriose was shown to be naturally released from actively growing plant tissue, and a treatment with pure thaxtomin A increased the amount of cellotriose exuded by radish seedlings [25]. On the other hand, cellobiose is the main product that results from cellulolytic degradation of dead plant material [27]. Our metabolomic analyses revealed that for most metabolites of the virulome, cellotriose was a stronger inducer than cellobiose (Fig. 1). Even for germicidins, one of the three metabolites whose production is reduced by cello-oligosaccharides, cellotriose had a stronger impact. One possible explanation is that, once internalized, cellotriose is hydrolysed to cellobiose and glucose by the beta-glucosidase BglC, therefore providing the best allosteric effector of CebR in the intracellular compartment. However, once inside the cell, the hydrolysis of cellobiose by BglC generates two molecules of glucose that will feed the glycolysis (primary metabolism) and no longer act as trigger for the specialized metabolism of *S. scabiei *. In contrast, our transcriptomic analysis suggested that cellobiose was in general a better elicitor compared to cellotriose (Figs. 3 and 4). This is not surprising as we showed that the import of cellobiose is faster than that of cellotriose in *S. scabiei* [28], which explains the observed slower transcriptional response. Also, RNA samples were collected after 1 h and 2 h post-addition of either cello-oligosaccharides while the extracts for the metabolomic analysis were collected after 96 h of growth. The short-term transcriptional response thus cannot be quantitatively compared to the long-term metabolite production response. Nevertheless, for the majority of BGCs and known metabolites investigated here, we saw a clear correlation between the data obtained via the transcriptomic and metabolomic approaches (Fig. S1). Among the exceptions is the case of the osmoprotectant ectoine. Its production was highly induced by both cello-oligosaccharides (Fig. 1) while there was no significant changes in gene expression (Fig. 3, Table 2). Similar as described above, the most plausible explanation lies in the fundamental difference in the culture conditions as samples for RNA-seq analyses were collected from liquid cultures just after the addition of the elicitors while metabolite samples were extracted from 96 h solid cultures. Osmotic protection is expected to be more important after 96 h cultures at the agar-air interface compared to a couple of hours in liquid cultures with no osmotic changes. The expression of genes involved in ectoine biosynthesis could also be controlled by development-related signals or regulators that are not yet available at RNA sampling time points. Finally, the transcriptional response of the BGC#20, BGC#28, and BGC#29 a for bottromycins, CFA-l-Ile, and germicidins, respectively, were too irregular (up-, or down-regulation depending on the elicitors and time points), or did not passed the threshold of statistical significance to make any correlation with the metabolomic study.

### CebR-independent response of most cello-oligosaccharide-dependent BGCs

Surprisingly, except for the thaxtomin gene cluster, only two of the 15 other BGCs that showed a strong transcriptional response to cellobiose and cellotriose also showed overexpression in the Δ*cebR* mutant (Fig. 5). This result, though unexpected, is in line with the absence of CebR-binding sites in the upstream region of the pathway-specific transcriptional activators and core biosynthetic genes of these cello-oligosaccharide expression-dependent BGCs. Through our earlier proteomic analysis, we also observed that many proteins in *S. scabiei* 87–22 whose production was activated by cellobiose did not show production changes in the *cebR* null mutant [50]. This could be explained by the possible ability of CebR to bind to ‘non-canonical’ DNA sequences, as previously observed for other transcription factors that also link nutrient sensing and the specialized metabolism [52]. Alternatively, cellobiose and cellotriose could be sensed by another and yet unknown transcription factor or thaxtomin itself could act as an intracellular trigger of the entire virulome.

### Cryptic metabolites and perspectives

Although strain *S. scabiei* 87–22 is well-studied as a model organism, the plurality of its cryptic and/or silent BGCs highlighted a huge reservoir of yet unknown metabolites. Nine of these cryptic BGCs showed a significant response to either both or one of the two cello-oligosaccharides, suggesting that some of these unknown compounds may also be part of the virulome of *S. scabiei *. The strongest transcriptional response was observed for BGC#32 (Table 2) involved in the synthesis of a siderophore type metabolite. Together with the transcriptional awakening of BGC#15, the cello-oligosaccharide-dependent response of siderophore-related BGCs further underlines the importance of iron acquisition during host colonization. We also observed increased expression for two BGCs responsible for the production of two bacteriocin type metabolites (BGC#7b and #13). The strain-specificity of these antibacterial peptides is unknown but their synchronized biosynthesis with other host colonization molecules could be seen as a strategy to prevent competing soil-dwelling bacteria to also access the starch reservoir of tubers.

The structure and the bioactivity of the metabolites whose production is triggered by cellobiose and cellotriose is currently under investigation and should lead to the identification of new key virulence determinants associated with the common scab disease.

## Supplementary Data

Supplementary material 1
