# Supplementary material for: The virulome of Streptomyces scabiei in response to cello-oligosaccharide elicitors
Source: Microb Genom. 2022 Jan 17;8(1):000760. doi: 10.1099/mgen.0.000760 (PMC8914351; doi:10.1099/mgen.0.000760)
Supplement: Supplementary material 1 [file mgen-8-0760-s001.pdf]

Supplementary Figure S1

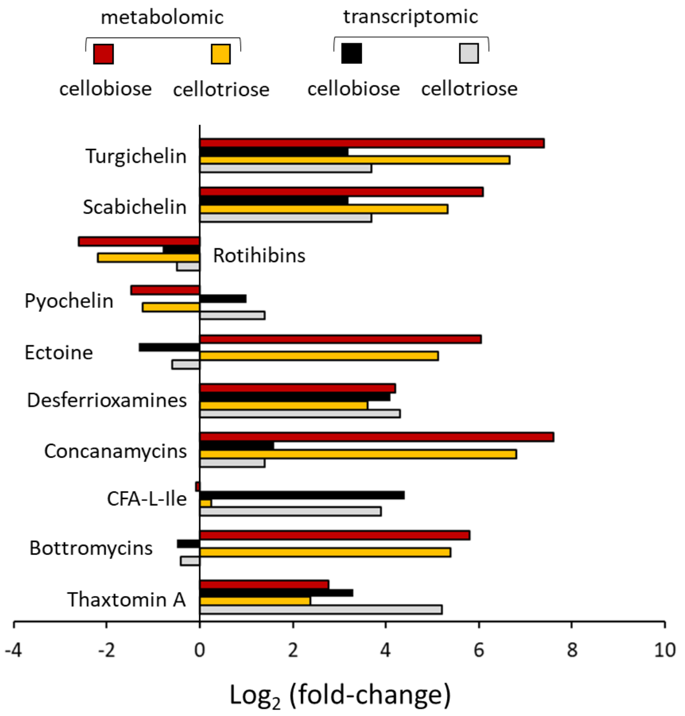

Figure S1. Correlation between Log2(Fold-change) of the transcriptomic and metabolomic analyses. Values for the production of bottromycins, concanamycins, desferrioxamines, and rotihibins are the mean of the values obtained for the different variants (see Figure 1).

Supplementary Figure S2

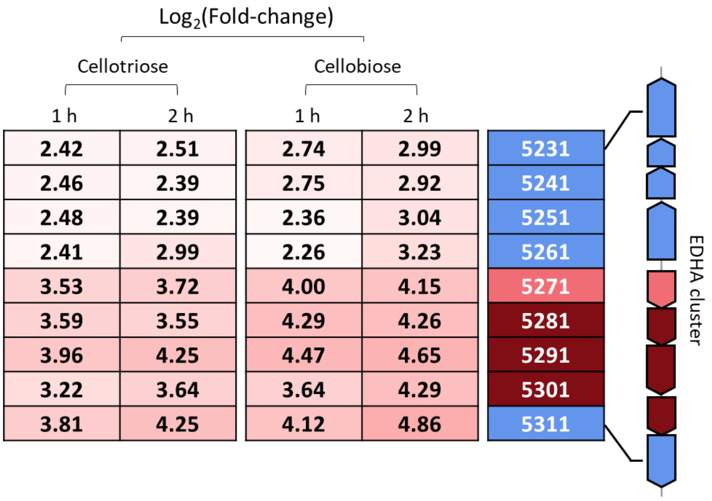

Figure S2. Detailed expression fold changes (expressed as Log2) for individual genes of the EDHAcluster in cellobiose and cellotriose after 1 or 2 hours of incubation. Gene color/function code: Blue, transport-related genes; Dark red, core biosynthetic genes; Pink: additional biosynthetic genes.

Supplementary Figure S3

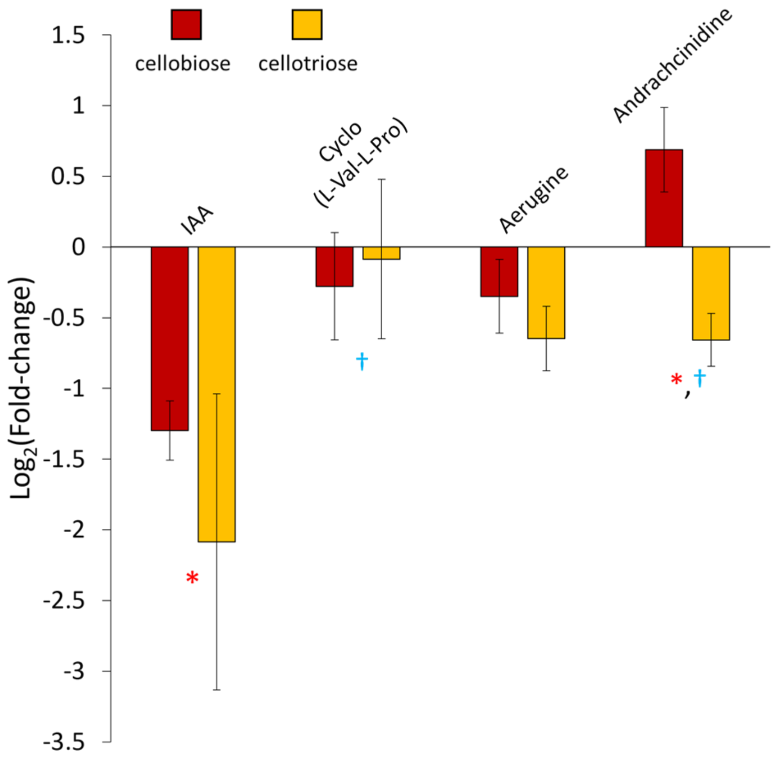

Figure S3. Log$_2$ of the fold-change observed in the production of the known specialized metabolites of *S. scabiei* 87-22 grown in the presence of cello-oligosaccharides. Bar plots display the Log2 fold-change of the mean of the normalized area under the curve of ion peaks detected in either cellobiose (red bars) or cellotriose (orange bars) culture conditions, against the mean of the normalized area under the curve of the same ion peak detected in the control "non-pathogenetic" sucrose conditions. The error bars display the standard deviation observed between three biological replicates (each with two to three technical replicates). †, indicates metabolites whose production was not statistically different in the sucrose condition compared to either cellobiose and/or cellotriose (p-values <0.05, bilateral t-Test). *, indicates metabolites whose production was statistically different between cellobiose and cellotriose.

Supplementary Table S1.

Table S1.
The MS conditions for the metabolites of interest in MRM mode.

Compound name	Parent (m/z)	Daughter ions (m/z)	Dwell (s)
Method 1			
Thaxtomin A	439.16	362.11 – 247.11 – 219.11	0.014
Concanamycin A	888.51	679.42 – 515.3 – 502.29 – 396.2 – 378.19 – 348.19 – 196.06	0.014
Concanamycin B	874.49	501.28 – 396.2 – 378.19	0.014
CFA-L-Ile	322.2	191.1 – 163.11 – 145.1 – 119.06	0.117
Desferrioxamine B	561.37	443.32 – 401.29 – 361.32 – 319.3 – 243.19 – 201.17	0.014
Desferrioxamine E	601.36	401.24 – 283.27 – 231.91 – 201.24	0.014
Scabichelin	648.37	518.28 – 346.2 – 259.65	0.014
Turgichelin	621.32	449.23 – 362.2 – 235.06 – 218.11	0.014
Pyochelin	325.06	189.98 – 172 – 145.95 – 127.96	0.117
Bottromycin A2	823.45	637.4 – 494.33 – 476.32 – 363.24 – 315.1	0.014
Bottromycin B2	809.45	623.4 – 480.31 – 462.33 – 349.22 – 315.1	0.014
Bottromycin D2	809.44	623.4 – 494.34 – 476.32 – 363.23 – 301.1	0.014
Bottromycin E2	795.41	609.37 – 466.31 – 448.3 – 349.24 – 315.1	0.014
Germicidin A	197.12	168.08 – 151.07 – 123.12 – 97.03 – 81.07	0.014
Germicidin B	183.1	168.08 – 137.1 – 109.1 – 67.05	0.014
Ectoine	143.08	125.06 – 120.02 – 102.09 – 97.08	0.014
Method 2			
Rotihibin C	860.62	742.55 – 594.48 – 551.36 – 450.31 – 310.28 – 285.2 – 249.16 – 202.15	0.051
Rotihibin D	874.61	756.55 – 608.48 – 551.36 – 450.31 – 324.28 – 285.2 – 249.16 – 202.15	0.051
IAA	175.7	129.9 – 102.9 – 77.1	0.105
Cyclo(L-Val-L-Pro)	197.13	70.07	0.296
Aerugine	210.06	120.04 – 91.02 – 73.01	0.051
Andrachcinidine	228.2	186.19 – 151.15 – 95.09	0.051

Supplementary Table S2.

Table S2.
List of the core biosynthetic gene(s) and their predicted function for each BGC

BGC	Old locus tag	Locus tag	Gene function	Specialized metabolite
1a	SCAB_1411	SCAB_RS00635	AMP-binding protein	Pyochelin
	SCAB_1471	SCAB_RS00665	Amino acid adenylation domain-containing protein	
	SCAB_1481	SCAB_RS00670	Non-ribosomal peptide synthetase	
1b	SCAB_1531	SCAB_RS00695	Fatty acyl-AMP ligase	Cryptic
	SCAB_1551	SCAB_RS00700	Fatty acyl-AMP ligase	
	SCAB_1671	SCAB_RS00760	AMP-binding protein	
2	SCAB_2011	SCAB_RS00900	AMP-binding protein	Cryptic
	SCAB_2041	SCAB_RS00915	Acyl--CoA ligase	
	SCAB_2071	SCAB_RS00930	4-hydroxy-2-oxovalerate aldolase	
	SCAB_2131	SCAB_RS00960	Long-chain fatty acid--CoA ligase	
3	SCAB_3221	SCAB_RS01465	Non-ribosomal peptide synthetase	Rothibins
	SCAB_3281	SCAB_RS01495	Fatty acyl-AMP ligase	
	NA	SCAB_RS01525	Non-ribosomal peptide synthetase	
4	SCAB_3621	SCAB_RS01675	Phosphotransferase	Cryptic
5	SCAB_5041	SCAB_RS02330	Terpene synthase family protein	2-methylisoborneol
6a	SCAB_5441	SCAB_RS02515	Phytoene/squalene synthase family protein	Isorenieratene
	SCAB_5491	SCAB_RS02535	NAD(P)-binding protein	
7a	SCAB_8611	SCAB_RS04055	Protein kinase/lanthionine synthetase C family protein	Informatipeptin
7b	SCAB_8681	SCAB_RS04085	Hypothetical protein	Cryptic
8	SCAB_12081	SCAB_RS05740	AfsA/ScbA-like protein	Cryptic
9	SCAB_12951	SCAB_RS06160	Squalene--hopene cyclase	Hopene
	SCAB_12991	SCAB_RS06175	Presqualene diphosphate synthase HpnD	
	SCAB_13001	SCAB_RS06180	Squalene synthase HpnC	
10	SCAB_18391	SCAB_RS08695	Iron transporter	Cryptic
	SCAB_18401	SCAB_RS08700	IucA/IucC family siderophore biosynthesis protein	
11a	SCAB_19691	SCAB_RS09315	Non-ribosomal peptide synthetase	Cryptic
12	SCAB_20121	SCAB_RS09510	terpene synthase family protein	Geosmin
13	SCAB_20761	SCAB_RS09810	DUF692 domain-containing protein	Cryptic
14	SCAB_23181	SCAB_RS10960	Di-trans,poly-cis-decaprenylcistransferase	Cryptic
15	SCAB_24681	SCAB_RS11650	Iron transporter	Cryptic
16a	SCAB_31781	SCAB_RS15080	Amino acid adenylation domain-containing protein	Thaxtomins
	SCAB_31791	SCAB_RS15085	Amino acid adenylation domain-containing protein	
16b	SCAB_32031	SCAB_RS15175	Lantibiotic dehydratase	Cryptic
	SCAB_32041	SCAB_RS15180	Lanthionine synthetase C family protein	
	SCAB_32051	SCAB_RS15185	Hypothetical protein	
17	SCAB_43301	SCAB_RS20600	Beta-ketoacyl-[acyl-carrier-protein] synthase family protein	Spore pigment
	SCAB_43311	SCAB_RS20605	Polyketide beta-ketoacyl synthase	
18	SCAB_43961	SCAB_RS20905	Hybrid non-ribosomal peptide synthetase/type I polyketide synthase	Cryptic
19	SCAB_47641	SCAB_RS22675	Protein kinase/lanthionine synthetase C family protein	Cryptic
20	SCAB_56611	SCAB_RS27005	RiPP maturation radical SAM protein 1	Bottromycins
	SCAB_56641	SCAB_RS27020	Alpha/beta hydrolase	
	SCAB_56651	SCAB_RS27025	RiPP maturation radical SAM protein 1	
	SCAB_56691	SCAB_RS27040	RiPP maturation radical SAM protein 1	
21	SCAB_57921	SCAB_RS27660	IucA/IucC family siderophore biosynthesis protein	Desferrioxamines
22	SCAB_59231	SCAB_RS28265	Tyrosinase co-factor	Melanin
23a	SCAB_62941	SCAB_RS30055	Acyltransferase domain-containing protein	Cryptic
23b	SCAB_63031	SCAB_RS30100	Hypothetical protein	Cryptic
	SCAB_63031	SCAB_RS30100	Hypothetical protein	
	SCAB_63111	SCAB_RS30140	Secondary metabolite corepressor	
	NA	SCAB_RS30155	SagB/ThcOx family dehydrogenase	
	SCAB_63151	SCAB_RS30160	TOMM precursor leader peptide-binding protein	
23c	SCAB_63111	SCAB_RS30140	Secondary metabolite corepressor	Cryptic
	NA	SCAB_RS30155	SagB/ThcOx family dehydrogenase	
	SCAB_63151	SCAB_RS30160	TOMM precursor leader peptide-binding protein	
	SCAB_63251	SCAB_RS30195	Secondary metabolite synthesis protein	
23d	SCAB_63111	SCAB_RS30140	Secondary metabolite corepressor	Cryptic
	NA	SCAB_RS30155	SagB/ThcOx family dehydrogenase	
	SCAB_63151	SCAB_RS30160	TOMM precursor leader peptide-binding protein	
	SCAB_63251	SCAB_RS30195	Secondary metabolite synthesis protein	
	SCAB_63281	SCAB_RS30210	Secondary metabolite biosynthesis protein	
24	SCAB_70721	SCAB_RS33840	Ectoine synthase	Ectoine
25	SCAB_72991	SCAB_RS34930	Formate dehydrogenase	Cryptic
26	SCAB_73741	SCAB_RS35290	Hypothetical protein	Cryptic
27a	SCAB_78961	SCAB_RS37805	Acyltransferase domain-containing protein	Cryptic
27b	SCAB_79221	SCAB_RS37920	Tryptophanase	Cryptic
28	SCAB_79651	SCAB_RS38130	Type I polyketide synthase	Coronafacoyl phytotoxins
	SCAB_79661	SCAB_RS38135	Type I polyketide synthase	
29b	SCAB_80171	SCAB_RS38390	Polyketide synthase	Germicidins
30	SCAB_82161	SCAB_RS39305	Cyclase	Cryptic
31a	SCAB_83871	SCAB_RS40110	Type I polyketide synthase	Concanamycins
	SCAB_83891	SCAB_RS40115	Type I polyketide synthase	
	SCAB_83901	SCAB_RS40120	Type I polyketide synthase	
	SCAB_83911	SCAB_RS40125	Type I polyketide synthase	
	SCAB_83921	SCAB_RS40130	Type I polyketide synthase	
	SCAB_83931	SCAB_RS40135	Type I polyketide synthase	
31b	SCAB_84181	SCAB_RS40250	Hypothetical protein	Cryptic
32	SCAB_84501	SCAB_RS40405	Siderophore biosynthesis protein	Cryptic
	SCAB_84511	SCAB_RS40410	Iron transporter	
33a	SCAB_85471	SCAB_RS40875	Non-ribosomal peptide synthetase	Scabichelin / Turgichelin
33b	SCAB_85691	SCAB_RS40985	Tyrosinase	Melanin
34	NA	SCAB_RS41210	Squalene/phytoene synthase family protein	Cryptic
